# Genome and life-history evolution link bird diversification to the end-Cretaceous mass extinction

**DOI:** 10.1126/sciadv.adp0114

**Published:** 2024-07-31

**Authors:** Jacob S. Berv, Sonal Singhal, Daniel J. Field, Nathanael Walker-Hale, Sean W. McHugh, J. Ryan Shipley, Eliot T. Miller, Rebecca T. Kimball, Edward L. Braun, Alex Dornburg, C. Tomomi Parins-Fukuchi, Richard O. Prum, Benjamin M. Winger, Matt Friedman, Stephen A. Smith

**Affiliations:** ^1^ Department of Ecology and Evolutionary Biology, University of Michigan, 1105 North University Avenue, Biological Sciences Building, University of Michigan, Ann Arbor, MI 48109, USA.; ^2^ Museum of Paleontology, University of Michigan, 1105 North University Avenue, Biological Sciences Building, University of Michigan, Ann Arbor, MI 48109, USA.; ^3^ Museum of Zoology, University of Michigan, 1105 North University Avenue, Biological Sciences Building, University of Michigan, Ann Arbor, MI 48109, USA.; ^4^ Department of Biology, California State University, Dominguez Hills, Carson, CA 90747, USA.; ^5^ Department of Earth Sciences, University of Cambridge, Downing Street, Cambridge CB2 3EQ, UK.; ^6^ Museum of Zoology, University of Cambridge, Downing Street, Cambridge CB2 3EJ, UK.; ^7^ Department of Plant Sciences, University of Cambridge, Downing Street, Cambridge CB2 3EA, UK.; ^8^ Department of Evolution, Ecology, and Population Biology, Washington University in St. Louis, St. Louis, MO 63130, USA.; ^9^ Department of Forest Dynamics, Swiss Federal Institute for Forest, Snow, and Landscape Research WSL, Zürcherstrasse 111 8903, Birmensdorf, Switzerland.; ^10^ Center for Avian Population Studies, Cornell Lab of Ornithology, Cornell University, Ithaca, NY 14850, USA.; ^11^ Department of Biology, University of Florida, Gainesville, FL 32611, USA.; ^12^ Department of Bioinformatics and Genomics, University of North Carolina at Charlotte, Charlotte, NC 28223, USA.; ^13^ Department of Ecology and Evolutionary Biology, University of Toronto, Toronto, Ontario M5S 3B2, Canada.; ^14^ Department of Ecology and Evolutionary Biology, Yale University, New Haven, CT 06520, USA.; ^15^ Peabody Museum of Natural History, Yale University, New Haven, CT 06520, USA.; ^16^ Department of Earth and Environmental Sciences, University of Michigan, 1100 North University Avenue, University of Michigan, Ann Arbor, MI 48109, USA.

## Abstract

Complex patterns of genome evolution associated with the end-Cretaceous [Cretaceous-Paleogene (K–Pg)] mass extinction limit our understanding of the early evolutionary history of modern birds. Here, we analyzed patterns of avian molecular evolution and identified distinct macroevolutionary regimes across exons, introns, untranslated regions, and mitochondrial genomes. Bird clades originating near the K–Pg boundary exhibited numerous shifts in the mode of molecular evolution, suggesting a burst of genomic heterogeneity at this point in Earth’s history. These inferred shifts in substitution patterns were closely related to evolutionary shifts in developmental mode, adult body mass, and patterns of metabolic scaling. Our results suggest that the end-Cretaceous mass extinction triggered integrated patterns of evolution across avian genomes, physiology, and life history near the dawn of the modern bird radiation.

## INTRODUCTION

Over 40 ago, Alvarez *et al.* (*1*) provided chemical evidence indicating that the Cretaceous-Paleogene (K–Pg) mass extinction was associated with an extraterrestrial impact. Subsequent research has refined our understanding of how this cataclysmic event influenced biodiversity [e.g., (*2*, *3*)]. Mounting evidence suggests that the K–Pg extinction event triggered convergent patterns of life-history evolution. For example, some lineages may have experienced a transient “Lilliput effect” in which average body sizes became smaller, likely through faunal sorting, dwarfing, or miniaturization (*4*, *5*). While great effort has been devoted to investigating extinction patterns among various groups across the K–Pg boundary [e.g., (*6*–*8*)], the impact of the end-Cretaceous mass extinction on the genomes of surviving lineages has received less attention.

Given that life-history traits such as body mass, generation length, and metabolic rates are linked to different aspects of molecular evolution (*9*), it is plausible that convergent patterns of life-history evolution across extinction boundaries impart distinct signatures in the genomes of surviving lineages (*10*–*12*). For example, in plants, repeated evolution of polyploidy may be associated with the K–Pg transition (*13*). Similarly, increased avian substitution rates may reflect extinction-related size-selectivity (*11*, *12*). Still, only a few studies have attempted to investigate how the aftermath of the K–Pg mass extinction shaped genome evolution [e.g., (*11*, *13*–*16*)]. We generally expect life-history evolution to influence phylogenetic patterns [e.g., (*17*)] because factors like effective population size (N_e_) and body mass are linked through environmental carrying capacity (*18*, *19*). The phenomenon of GC-biased gene conversion also appears to have an important role in driving patterns of avian base composition (*20*, *21*), but this has never been directly linked to the K–Pg transition. Such a link might be expected, however, because of the relationships among life history, N_e_, recombination, and the efficacy of gene conversion (*21*, *22*).

Many studies attempting to connect events in Earth’s history to patterns of genome evolution rely on inferences from molecular clock analyses [e.g., (*11*, *13*)]. These approaches can reveal heterogeneous patterns in the tempo of molecular evolution [e.g., (*10*, *23*)] but typically assume that the underlying sequence data evolved according to the expectations of a homogeneous nucleotide substitution model. If this assumption is violated, time-homogeneous models may obscure important evolutionary patterns [e.g., (*17*)]. Nevertheless, techniques that enable substitution models to vary across a clade’s evolutionary history have not yet seen widespread adoption in the macroevolution literature [e.g., (*24*–*26*)]. Detecting where one model has shifted to another on a phylogeny may provide evidence of evolutionary transitions in the “mode” or process that generated the observed data (*23*, *27*–*29*). Thus, investigating patterns of model shifts across both genome and life-history traits may reveal unknown links among Earth’s history and evolutionary processes.

Here, we combine approaches from molecular systematics and phylogenetic comparative methods to investigate molecular model heterogeneity across the avian tree of life. We apply a novel stepwise approach to estimating the phylogenetic position of shifts in molecular substitution model parameters, implemented in Janus (Materials and Methods) (*30*). Our approach relaxes the assumption that the sequence data–generating process has remained constant through evolutionary time, enabling us to test the hypothesis that the radiation of birds near the end-Cretaceous extinction was accompanied by concurrent diversification in the mode of molecular evolution. Specifically, our phylogenomic analysis focused on evolutionary shifts in base composition, a well-established proxy of avian genome architecture (*21*, *30*–*32*). After inferring molecular shifts, we applied a random forest machine learning classifier to survey the organismal traits associated with inferred shifts. We also applied multivariate Ornstein-Uhlenbeck (OU) models (*27*–*29*) to investigate the hypothesis that molecular shifts co-occur with changes in the adaptive landscape or evolutionary allometries of key traits. We assessed aspects of breeding ecology, development, senescence, and metabolism that may have undergone intense selection or relaxation of evolutionary constraints during the K–Pg transition [e.g., (*10*, *11*, *33*–*35*)]. Model shifts across many dimensions of biodiversity were constrained to clade originations temporally associated with the K–Pg transition, linking patterns of genomic variation to life history, physiology, and macroevolutionary patterns detected from the fossil record.

## RESULTS

### Molecular model shifts

**Fig. 1.**
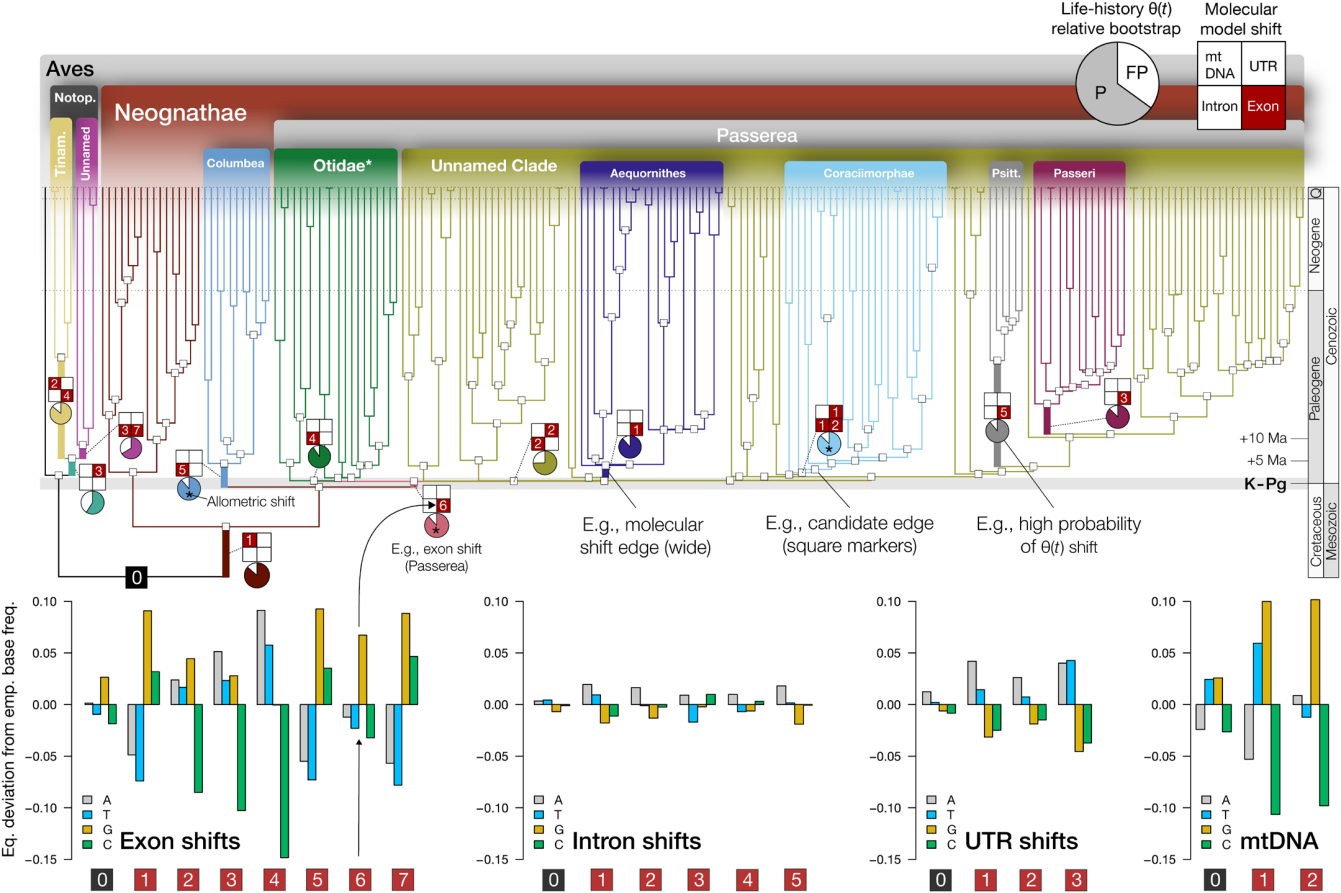
Inferred model shifts across phylogenomic and life-history data. Thirteen phylogenetic regimes encompassing 17 molecular model shifts were required to explain heterogeneity in equilibrium base frequencies across genetic data types (branches with distinct colors). Fifteen shifts were inferred at nodes with stem ages within ~5 Ma of the K–Pg boundary (*39*). (**Top**) The aggregate signal of molecular model shifts across nuclear and mitochondrial data types identified by Janus, mapped onto the MRL3 supertree, with the ancestral regime “0” in black [*Otidae = Otidimorphae + Strisores, (*150*)]. Patterns of molecular model shifts across phylogenetic edges are summarized as 2 × 2 grids (see legend above; figs. S3 and S7). Numeric labels at each grid position correspond to a molecular shift in a specific data type. Pie charts summarize the detection rate (“P”) for shifts in trait optima θ(*t*) across eight life-history traits relative to a simulated null false-positive (“FP”) rate {e.g., ℓ1ou detection rate / [ℓ1ou detection rate + false-positive rate] (statistical precision), under AICc; see fig. S4A}. (**Below**) Estimated magnitude of shifts in equilibrium base frequencies relative to the empirical base frequencies for a given taxon partition for each data type, ordered by dataset size (Discussion and fig. S7, A to D). Edges with well-supported shifts in metabolic allometry are labeled with an asterisk, with the most substantial support observed for Coraciimorphae (*pp* = 98%, Fig. 3).

Using a dataset spanning 198 avian lineages and 910 loci across coding and noncoding regions (Supplementary text), we inferred 17 molecular model shifts on 12 phylogenetic edges. Of these, 15 shifts were very close to the K–Pg boundary (Fig. 1 and figs. S1 and S7, A to D) (Materials and Methods) (*36*–*39*). Considering multiple shifts detected on the same edges, Janus inferred 13 phylogenetic regimes (one ancestral + 12 derived) are required to explain heterogeneity in sequence composition across exons, introns, untranslated regions (UTRs), and mitochondrial DNA (mtDNA), relative to the reference topology [“MRL3” in Kimball *et al.* (*39*); Fig. 1]. We emphasize that Janus does not rely on information about the absolute timing of divergence events (Materials and Methods); it is, therefore, notable that shifts cluster in close temporal proximity on a well-justified time-calibrated phylogeny (Fig. 1 and fig. S1) (*39*). Extensive simulations (*n* = 9200) conditioned on the shape of the avian phylogeny show low false-positive and false-negative rates (Materials and Methods and fig. S9).

Inferred molecular shifts were largely concordant with the origins of diverse ancient clades previously recognized as ordinal or superordinal taxonomic ranks. These include Notopalaeognathae, Tinamiformes, the unnamed sister clade to Tinamiformes (in the MRL3 tree; Rheiformes, Casuariiformes, and Apterygiformes), Neognathae, Columbea, Passerea, Otidae [i.e., Otidimorphae + Strisores, sensu (*36*)], the unnamed sister clade to Otidae (in the MRL3 tree: other Neoaves), Aequornithes, Coraciimorphae, Psittaciformes, and Passeri [table S1; also see (*12*, *32*) with respect to Passeri]. For every case, our approach inferred molecular shifts with 100% model weight when considering a shift’s existence and phylogenetic position, indicating that shifts have a strong statistical signal. Inferred molecular shifts often fall on the GC-AT axis (Fig. 1 and fig. S7, A to D) of nucleotide compositional variation, with most shifts occurring in our large exon dataset, followed by introns, UTRs, and mtDNA. There was no trend relating dataset size to the magnitude of inferred substitution parameters (Fig. 1, bottom). Sequence type best explains the relative deviation between estimated equilibrium and empirical base frequencies (e.g., coding versus noncoding; Fig. 1 and Discussion). We also find strong correspondence between a proxy of N_e_ and GC content across groups identified by Janus (fig. S8), similar to Weber *et al.* (*21*). These patterns appear robust to variation at individual codon positions in exons (Materials and Methods).

### Life-history evolution

**Fig. 2.**
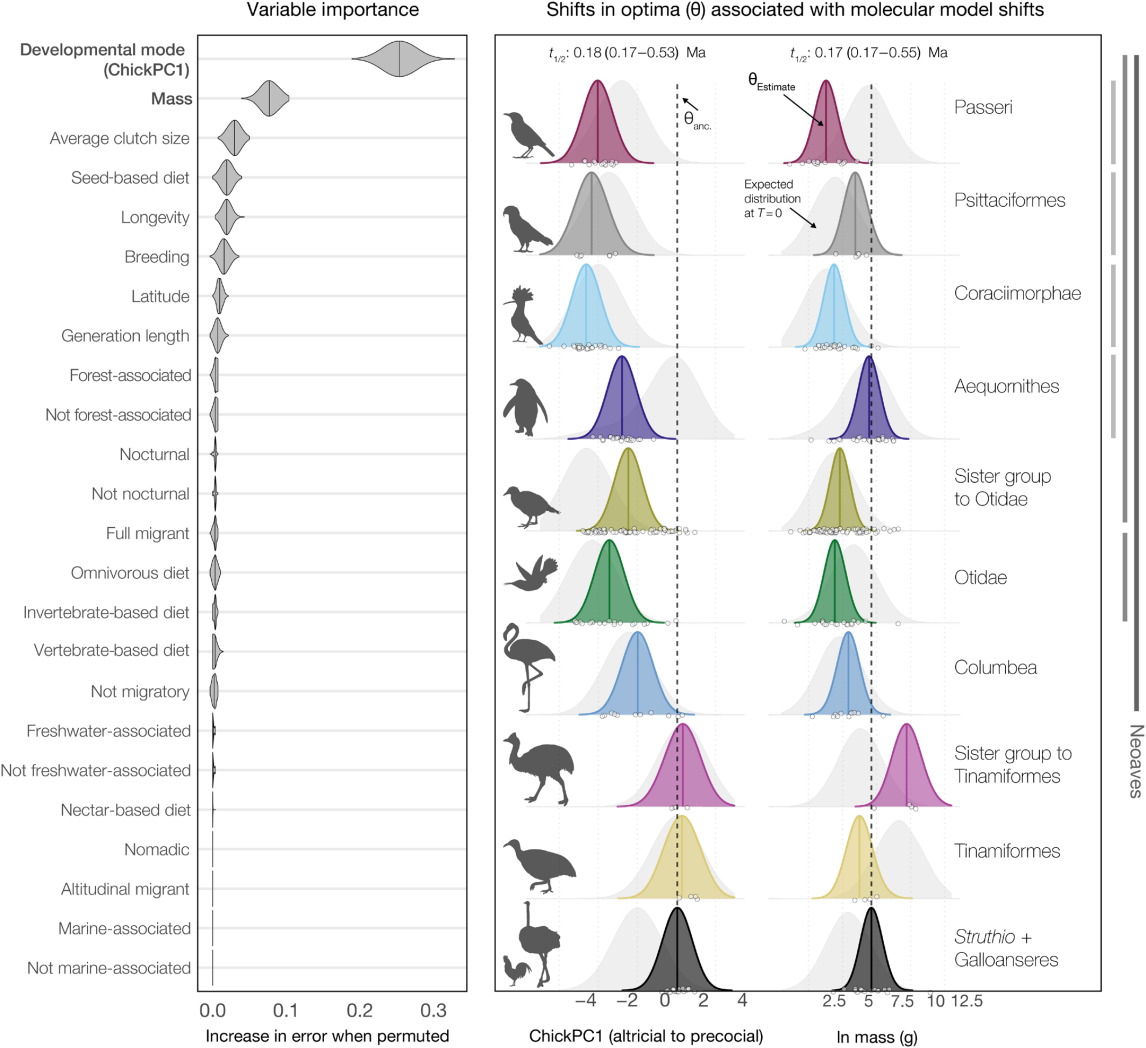
Life-history traits associated with inferred molecular model shifts. (**Left**) Permutation-based variable importance for life-history traits (*40*, *59*). With a random forest classifier, we identified variation in avian developmental mode [ChickPC1 in (*41*)] and adult body mass (a proxy of N_e_) as closely associated with taxon partitions recognized by Janus in an analysis of exon data (Materials and Methods). (**Right**) Estimates of trait optima θ(*t*) constrained to molecular model shift points from nuclear genetic data (OUM model, 100 parametric bootstraps; colors and labels match; Fig. 1). Background distributions (light gray) indicate simulated trait values at the present (e.g., expected values under the fitted model; diagnostic of model adequacy). Vertical bars (right) mark phylogenetic groups within Neoaves temporally associated with the K–Pg extinction (*12*, *37*, *39*). Molecular model shifts are generally associated with shifts toward increased altriciality or decreased adult body mass (also see figs. S5 and S6).

We applied a random forest machine learning approach to predict phylogenetic regimes inferred by Janus using a suite of candidate traits (Fig. 2 and Materials and Methods). Developmental mode (*40*), followed by adult body mass, were consistently the most important traits [area under the receiver operating characteristic curve (AUC) = 0.94] associated with estimated molecular shifts. Traits reflecting substrate or dietary preferences were relatively unimportant, except for granivory, which ranked fourth after average clutch size (Fig. 2 and figs. S5 and S6). In parallel, we explored the hypothesis that molecular model shifts coincide with shifts in the evolutionary optima of life-history traits (Materials and Methods and Figs. 1 and 2) using multi-optimum OU models (*28*). Under this framework, modeled optima [θ(*t*)] represent equilibrium points that a lineage’s traits evolve toward under the combined influence of stabilizing selection and genetic drift. For OU models that considered molecular shift points as candidates for θ(*t*) shifts, model precision was consistently high (Fig. 1 and fig. S4A): All molecular shift points were associated with optima shifts under alternative information criteria (e.g., 76.2 to 87.2%; see Supplementary text). Unconstrained analyses were also closely congruent with molecular shift points (Supplementary text and fig. S4B).

Molecular model shifts were broadly associated with θ(*t*) shifts toward increased altriciality at hatching or decreased adult body mass relative to θ_anc._, the ancestral optimum (within Neoaves, 7 of 7 and 6 of 7, respectively; Fig. 2). Aequornithes and Psittaciformes showed derived increases in body mass optima, along with derived shifts toward increased altriciality (Fig. 2, right) while also indicating an overall lower optimum than θ_anc_. Outside Neoaves, developmental mode optima within Palaeognathae were not clearly associated with molecular model shifts. For body mass, however, θ(*t*) for Tinamiformes was similar to θ_anc._, while its unnamed sister clade (Rheiformes, Casuariiformes, and Apterygiformes) showed a marked increase in θ(*t*) relative to θ_anc._ (Fig. 2). An alternative set of analyses estimating θ(*t*) separately for Struthio + root suggested all molecular shifts, including those within Palaeognathae, were associated with derived decreases in body mass or increases in altriciality (fig. S6 and Supplementary text).

### Metabolic allometry

**Fig. 3.**
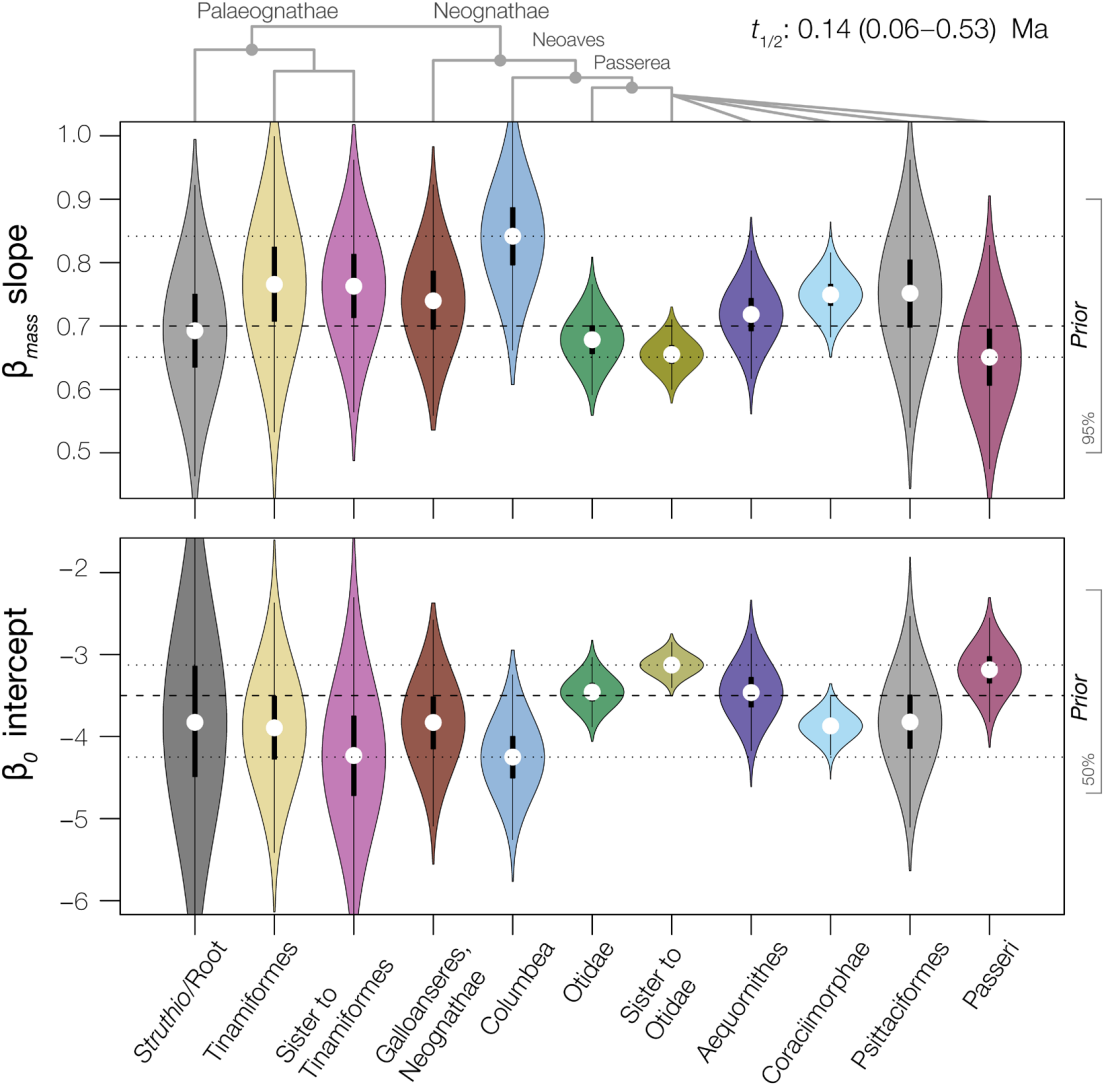
Inferred molecular model shifts are associated with a range of avian metabolic allometries. We analyzed metabolic data under a Bayesian framework to generate posterior estimates of intercept (β_0_) and slope (β_mass_) coefficients from an evolutionary allometric regression model. Here, we depict the posterior estimates for slope and intercept across a fixed allometric shift model recapitulating molecular model regimes across all data types (single branch internodes reflecting Passerea and Notopalaeognathae not shown; see table S1 and fig. S10). Modal parameter estimates are indicated with white dots. Horizontal dashed lines indicate the prior mean; dotted lines indicate the range of modal parameter estimates. On the right axis, we show 95% (for slope) and 50% (for intercept) prior density intervals. Our results show that a shift toward a lower slope and higher intercept characterizes lineages originating near the end-Cretaceous transition (e.g., Passerea).

Across the Tree of Life, organism mass and metabolic rate broadly follow a three-fourth power scaling law [e.g., (*41*)]. We applied a Bayesian model of metabolic scaling [Materials and methods; (*29*)] and found that deviations from three-fourth scaling are associated with inferred molecular model shifts close to the K–Pg boundary. Modal estimates for slope (β_mass_) ranged from 0.65 (~⅔) to 0.84 (~⅘) and intercept (β_0_) from −4.25 to −3.13 (Fig. 3 and table S1), similar to estimates for avian and mammalian subclades (*42*, *43*). Compared to θ(*t*) for life-history traits like mass or developmental mode (Fig. 2 and figs. S5 and S6), metabolic scaling parameters have more uncertainty [e.g., Palaeognathae; Fig. 3 and fig. S10]. Modal posterior estimates (Fig. 3) indicate that the origins of K–Pg–associated subclades within Neoaves coincide with a shift toward overall lower body mass (Fig. 2 and figs. S5, S6, and S10), as well as lower slope and higher intercept terms [Fig. 3 and fig. S10; e.g., under 10 kg as noted in (*42*, *44*)]. Derived shifts in metabolic scaling followed patterns we identified for body mass (Fig. 2 and fig. S10), with decreased mass leading to weaker metabolic scaling (*29*, *45*). Seven edges detected at a 10% posterior probability cutoff reflect K–Pg–associated clade originations (Fig. 3 and table S1). Under a more conservative threshold, only three candidate edges were detected with moderate to strong support, including the unnamed sister clade of Otidae (*pp* ~ 38%), Columbea (*pp* ~ 39%), and Coraciimorphae (*pp* ~ 98%) (Fig. 1). Notably, the diverse clade Coraciimorphae was the only group for which molecular model shifts were detected across all nuclear genetic data types. Overall, metabolic parameter estimates were consistent with the hypothesis that allometric shifts in avian metabolism are associated with molecular model shifts near the K–Pg boundary.

## DISCUSSION

Unraveling interactions among significant events in Earth’s history and macroevolutionary patterns is a fundamentally important yet persistently challenging goal in evolutionary biology. Here, we first investigated how the adaptive radiation of birds near the K–Pg boundary is linked to patterns of molecular evolution. We show that temporal proximity to the K–Pg boundary increases the probability of molecular model shifts (Fig. 1 and fig. S1, A to D), linking a major mass extinction to macroevolutionary changes in the mode of avian genome evolution. By anchoring a series of phylogenetic comparative models with shifts in nucleotide composition, we then find evidence that shifts in genome evolution were likely concurrent with shifts in the evolutionary optima θ(*t*) of important avian life-history traits (Fig. 2 and fig. S4, A and B), as well as shifts in metabolic allometric slope β_mass_ and intercept β (Fig. 3). Broadly, our estimates of model shifts in genomic sequences coincided with shifts toward increased altriciality or smaller adult body mass, consistent with the hypothesis of a K–Pg–associated Lilliput effect [e.g., (*4*, *8*, *11*, *12*)].

Our examination of metabolic allometry provides insights into the consequences of size evolution. Transitions toward smaller sizes are correlated with weaker scaling relationships between metabolic rate and body mass [e.g., along the Neoaves-Passerea topology; Fig. 3 and fig. S10; (*11*)]. This pattern implies that the energetic costs of evolutionary increases in size are reduced in clades with a smaller average body mass. In the aftermath of the K–Pg extinction, in which networks of ecological competition were reset, the survivorship of clades with smaller body sizes—and weaker associations between metabolic rate and body mass—may therefore have facilitated the evolution of variable physiological strategies in the early Cenozoic [e.g., (*46*, *47*)]. This deduction is consistent with theoretical and empirical advances that predict that transitions toward harsher environments with increased extrinsic mortality drive the evolution of lower slope values in metabolic scaling relationships (*41*). These phenomena are associated with earlier maturation and faster growth (*41*), aligning with our inference of increased altriciality associated with the K–Pg extinction [e.g., (*40*, *48*)].

### Recognizing early bursts

Lineages can enter novel adaptive zones during diversification if ecological, geographic, or phenotypic opportunities arise [e.g., (*49*, *50*)]. The aftermath of mass extinctions, especially those of short duration, may present all three classes of opportunities, resulting in recovery faunas that experience “early bursts” of lineage and character diversification (*51*, *52*). If diversification becomes constrained as niches fill [e.g., (*53*)], rates of morphological evolution and lineage accumulation should decline, with the fastest rates of change restricted to a short interval following the mass extinction event (*54*, *55*). Accordingly, we expect initially high rates of evolution to generate outsized disparity (e.g., trait variance or heterogeneity) early in post-extinction adaptive radiations.

An exclusive focus on rates of change, however, [e.g., (*56*)] may obscure other kinds of early burst patterns [e.g., (*57*)]. Our approach diagnoses a “molecular early burst” in which disparate patterns of nucleotide sequence evolution arose within a relatively short interval near the K–Pg boundary. Conceptually, this approach is more similar to paleontological approaches [e.g., (*57*, *58*)] than it is to techniques that estimate rates of change in quantitative or molecular characters [e.g., (*11*, *56*)]. While many studies have quantified early bursts through patterns of morphological evolution or rates of lineage diversification, we show that ancient diversification events may impart a signature of increased genomic disparity that remains detectable in surviving lineages for tens of millions of years. In this context, the molecular model shifts we infer may represent “genomic fossils” associated with canalized macroevolutionary regimes.

### A novel dimension of avian adaptive radiation

Our inference of numerous model shifts within an ~5 Ma interval of the K–Pg boundary (Fig. 1) supports an “early burst” mechanism in which ecological and lineage disparity accumulated rapidly in the early history of crown birds. Given that the uncertainty in estimated molecular divergence times typically exceeds 5 Ma (*11*, *36*–*39*), a conservative interpretation of available divergence time estimates does not reject the hypothesis that these events were closely linked (Fig. 1 and fig. S1).

Consistent with this pattern, models of quantitative trait evolution estimate short phylogenetic half-lives [(*t*_1/2_ = ln(2)/α, e.g., *t*_1/2_ body mass ~0.18 (0.17 to 0.52) Ma; Fig. 2]. Such short intervals imply rapid character change followed by a relatively stationary process [a median generation length of 3 years (*59*) suggests a *t*_1/2_ of ~60,000 generations]. This interpretation is consistent with our current understanding of the avian fossil record, which indicates only limited crown bird diversification in the Late Cretaceous [e.g., (*10*, *11*, *38*)]. Our results, therefore, support the hypothesis that developmental and life-history traits were canalized early in crown bird evolutionary history (*60*), partitioning higher-taxa into distinct types [e.g., (*57*, *61*, *62*)]. Considering the association between inferred molecular model shifts and shifts in the evolutionary optima of life-history traits (e.g., fig. S4, A and B), both patterns indicate integrated evolutionary changes [e.g., (*57*)] to the post-Cretaceous adaptive landscape and permanent shifts to new adaptive zones arising in the early Cenozoic [e.g., (*50*)].

While many mechanisms link substitution rates to the life-history spectrum (*9*), we have less intuition about how the mode of molecular evolution may relate to life-history variation. One idea proposed to explain variation in DNA compositional heterogeneity links the recombination process of GC-biased gene conversion to generation length, N_e_, and covarying life-history traits [e.g., (*21*, *22*, *30*, *63*, *64*)]. Post-Cretaceous increases in N_e_ (*12*, *14*) and decreased generation lengths (*11*) may, therefore, contribute to the patterns we observe, as higher N_e_ is predicted to increase the efficacy of GC-biased gene conversion (fig. S8) (*21*). Further, most inferred substitution model shifts occur in exon data, a pattern consistent with the hypothesis that transcriptionally active regions with elevated rates of recombination may be more subject to GC-biased gene conversion. Birds lack the DNA binding protein PRDM9, which, in mammals, directs recombination away from transcriptionally active regions (*65*). Thus, our observation that coding regions show greater deviations between estimated equilibrium and empirical base frequencies could be influenced by model fit related to functional constraints, codon usage bias, recombination, or selection (see supplementary analysis of codon usage; figs. S2 and S7, A to D).

Notably, our study suggests model heterogeneity as a mechanism to explain the “data type” effect (*12*, *36*, *66*–*68*), in which phylogenetic analyses of coding or noncoding sequence data recover conflicting signals of avian phylogeny. A recent analysis of whole genomes (*12*) did not recover several clades on which we detect shifts in base frequencies (e.g., Columbea and Passerea), suggesting that model heterogeneity may have important consequences for phylogenetic topology inference. We also expect that, in cases where homogeneous models are used, shifts in substitution parameters could lead to biased estimates of branch lengths, potentially contributing to older estimates of divergence dates inconsistent with the fossil record [e.g., (*10*, *11*)]. Nevertheless, our results support recent inferences (*12*, *66*, *67*) that noncoding introns or intergenic regions may be preferable for inferring avian phylogeny because exons exhibit more heterogeneous evolutionary dynamics.

In conclusion, although high-throughput sequencing has clarified the evolutionary history of many vertebrate clades, the early diversification history of crown birds—a group comprising more than 10,000 extant species—continues to provoke debate. Here, we used nonhomogeneous models of sequence evolution to investigate how the diversification of modern birds was marked by shifts across many axes of natural variation. Our results suggest that directional selection on key parameters across the end-Cretaceous mass extinction event—favoring traits such as increased altriciality or reduced adult body mass—may have shifted patterns of genome evolution through their linkages with population-level and demographic processes. Overall, our findings support the hypothesis that one of the most significant events in the history of life on Earth–the Chicxulub bolide impact and its associated mass extinction at the end of the Cretaceous Period–catalyzed an integrated evolutionary response within surviving lineages, ultimately giving rise to the spectacular diversity of living birds.

## MATERIALS AND METHODS

### Nuclear sequence data collection and processing

We reassembled an existing short-read sequence targeting 394 gene regions across 198 bird species and two crocodilian outgroups from Prum *et al.* (*37*). These data were initially collected using target-capture of anchored hybrid enrichment loci (*69*), a set of single-copy regions semiconserved across vertebrates. We analyzed the existing raw sequencing reads with a common pipeline designed to extract phased exons. First, we removed low-quality regions and adaptor sequences using Trimmomatic v0.36 (*70*) and merged overlapping reads using FLASH v1.2.11 (*71*). We assembled reads for each sample using Trinity v2.11 (*72*). We then annotated assemblies by comparing assembled contigs to target loci using blat v36x2 (*73*). To ensure that we annotated orthologs, we retained only contigs with a reciprocal best-hit match to a target locus. To identify intron-exon boundaries, we used exonerate 2.4.0 to compare the nucleotide sequences of annotated loci to the protein sequences for the exons of each locus based on protein-coding data and annotations from the zebra finch (*Taeniopygia guttata*, genome assembly bTaeGut1_v1). This approach assumes that intron-exon boundaries are conserved across the avian radiation. Occasionally, mapping of the exon sequence to the nucleotide sequence was discontinuous, suggesting the presence of an intervening noncoding region. In such cases, we retained the highest-scoring contiguous stretch of sequence only.

To identify variable sites, we mapped cleaned reads back to annotated contigs using bwa v0.7.17-r1188 (*74*) and used GATK v4.1.8 to mark duplicates (*75*). We called variants on this alignment using GATK HaplotypeCaller and filtered it to only retain variants with coverage >20× and quality >20. Using this high-quality variant set, we recalibrated the base quality scores in the alignment files using GATK. We then called the variants and phased them using HaplotypeCaller. Last, we exported diplotypes and phased haplotypes per intron and exon in a coding region, masking any sites with coverage <2×. Ultimately, we captured 453 exons, 573 introns, and 213 UTRs. Before alignment, we applied a series of sequential filtering steps to remove the remaining short or low-quality fragments. We removed (i) leading and trailing N characters from each fragment and the resulting sequences that were zero length (see locus-filtering.R script), (ii) fragments with >40% N characters, (iii) fragments that were <50–base pair (bp) long, and (iv) whole loci that lacked coverage for at least 10% of the taxa in the dataset.

We aligned phased exon sequences with the Multiple Alignment of Coding Sequences (MACSE) ALigning, Filtering, and eXporting pipeline (ALFIX) (*76*). MACSE-ALFIX chains together several programs that perform reading frame aware alignment with MACSE and subsequent alignment filtering with HmmCleaner (*77*) to remove nonhomologous sequence fragments. We aligned phased noncoding sequences with Fast Statistical Alignment (FSA) (*78*). We calculated alignment statistics using AMAS (*79*). We used trimAl (*80*) to evaluate the effect of 5 to 30% alignment column occupancy filtering on alignment length and the loss of parsimony informative sites. We ultimately filtered our noncoding alignments to require a minimum column occupancy of 5% (i.e., 95% of the sequences in an alignment are allowed to contain a gap for a given site). This procedure, which we believe is conservative, increased the signal-to-noise ratio in these data by removing stretches of unaligned nucleotides (characteristic of FSA alignments) while retaining most of the informative data (*81*, *82*). Unfiltered alignments and the final filtered dataset are provided as Supplementary Data.

### Mitochondrial sequence data collection and processing

We ran Mitofinder 1.4 (*83*) to identify the mitochondrial regions from the previously assembled contigs. For reference mitogenomes, we used complete mitogenomes available in GenBank (table S3). When available, we used a reference from the same order [although for Passeriformes, we used different references for oscines (Passeri) and suboscines (Tyranni)]; in a few cases, it was necessary to use a reference from a closely related order (table S3). We then extracted the 13 protein-coding genes and 2 ribosomal RNAs (rRNAs) from the mitofinder output (final_genes.fasta file). In some cases, limited mitochondrial data were recovered (table S3). In those cases, we searched GenBank for the same or a phylogenetically equivalent species that could be substituted. When no suitable alternative was available from GenBank, we also used mitogenomes assembled from the raw data collected as part of other studies (*84*–*88*). To increase data coverage in five cases, we generated chimeric sequences using available GenBank data from multiple individuals of the same species (table S3).

Once a set of sequences had been assembled, we performed an initial analysis using these data, combined with a larger set of mitogenomes to ensure sequences were correctly identified (placed phylogenetically with expected relatives) and did not exhibit unusually long-branch lengths, which might suggest assembly errors. To do this, we ran an initial alignment using MAFFT 7.294b (*89*) using default parameters. This alignment was then analyzed in IQ-TREE 2.1.2 (*90*) using the GTR + I + G4 substitution model with 1000 ultrafast bootstrap replicates (*91*). Last, we regenerated alignments for the present study using the same procedure described above for nuclear coding and noncoding data.

### Phylogenetic frameworks

To avoid issues of circularity related to inferring molecular patterns and phylogenetic topology from the same molecular dataset and to control for stochastic resolutions of Neoaves [e.g., (*92*)], our focal analyses use the MRL3 supertree [“MRL_3backbone”] (*39*) as a topological constraint. This topology balances the signal of phylogeny among several recently published avian genomics datasets and resolves the seven major higher-level clades identified by Reddy *et al.* (*66*), as well as the most robustly supported intraordinal clades (*12*, *37*). It is also in line with a growing number of studies that have suggested that early diversification events within the avian crown group were associated with the K–Pg boundary (*12*, *36*–*39*). As inference of avian phylogeny is an active area of research (*67*), we explored how patterns of gene-tree discordance [e.g., (*36*)] may confound inference of molecular model shifts or potential statistical associations with the K–Pg boundary (Supplementary text and fig. S1B).

Substitution model shift analysis with Janus takes a rooted input phylogram with branch lengths in substitution units [details below; (*30*)]. To generate starting trees for Janus, we used our reprocessed datasets and estimated maximum likelihood branch lengths with IQ-TREE v 2.1.1 (*90*, *93*). For each data type, we applied an optimal partition model selected with the MFP+MERGE approach in IQ-TREE (*94*, *95*), with each locus defined as the unit for partitioning. We estimated molecular branch lengths separately for exons, introns, UTRs, and mtDNA, but kept the topology fixed across datasets.

The Janus algorithm fits models that describe patterns of substitutions irrespective of the absolute timing of divergence events (below). Therefore, temporal patterns must be evaluated on a reference timeline. To interpret our model-shift results on a time-calibrated phylogeny, we used congruification (*96*) with treePL (*97*) to apply the divergence date estimates from the reduced taxon set analysis presented in (*39*) to the phylogenetic branch length estimates derived from the present study. The well-constrained divergence estimates from (*39*) are broadly congruent with those reported across several phylogenomic analyses of independent datasets (*12*, *36*–*38*). These estimates reject the hypothesis that many modern avian clades originated in the Cretaceous and center the diversification of most superordinal variation within ~±5 Ma of the K–Pg boundary (Fig. 1). Therefore, our interpretations are conditional on this general divergence time scenario, an area of active research [e.g., (*10*, *11*, *38*)] (see Discussion).

### Fitting time-heterogeneous models to avian phylogenomic data

For nuclear genomic data, we considered the signal across three concatenated datasets (exons, introns, and UTRs). For mitochondrial data, we considered three alternative datasets (all data combined, protein-coding genes combined, and rRNAs combined). Our focus on data type mirrors recent developments implicating this axis of genomic variation as a primary source of phylogenetic incongruence (*12*, *36*, *66*, *67*). We fit time-heterogenous substitution models to each dataset using Janus (commit 8952e31d, https://git.sr.ht/~hms/janus). Although Janus can search for shifts in base frequencies as well as the substitution rate matrix, initial explorations of our data indicated that shifts in the substitution rate matrix were negligible, suggesting that the primary axes of model heterogeneity in these data are related to base frequencies. Enabling a free rate matrix also dramatically increased the number of parameters and computing time. As a result, we only considered shifts in base frequencies for subsequent analyses.

We set each search to accommodate rate heterogeneity across sites according to a discretized gamma distribution (-g) and to assess model weights for the existence (-u) and location (-l) of model shifts. Simulations indicate that this combination of parameter options has high power (e.g., a negligible false-positive rate) to detect the phylogenetic position of molecular model shifts [details below; (*30*)]. Considering our genetic dataset’s taxonomic sample, we set the minimum clade size to >| = 4 (-m 4). Thus, the set of possible shift configurations reflects 101 internal nodes spanning ~77 Ma [square markers in Fig. 1; e.g., with postorder traversal, any node (excluding the root) with >| = 4 descendant edges].

### Implementation of Janus and the greedy search algorithm

Janus has been implemented in both Golang and C, and the source code is available at https://git.sr.ht/~hms/janus and https://git.sr.ht/~hms/hringhorni. The algorithm to detect shifts in stationary frequencies follows a stepwise procedure similar to Alfaro *et al.* (*98*) and Mitov *et al.* (*99*) and requires a rooted tree and matching alignment as input: 1) estimate a maximum likelihood root composition; 2) traverse the tree in postorder fashion (i.e., from the tips to the root) and estimate maximum likelihood compositions for subtrees with a minimum number of tips specified by the user; 3) take the subtree composition and the root composition for the remainder of the tree and estimate a likelihood and then a Bayesian Information Criterion (BIC) score (*100*); 4) order these compositional shift models for every eligible subtree by BIC; 5) initiate the final model configuration with the root model; then, in a greedy manner, add a shift to the root model based on the previously ordered subtrees, estimate a new BIC, and add the submodel to the set of models if the new BIC is lower; and 6) discard a submodel if the updated BIC score is increased. Then, using two approaches, we assess the relative support for shifts both with respect to a shift’s existence and its location. First, we assess shift existence (-u flag) by evaluating BIC weights for alternative models that include or exclude each proposed shift. Next, we assess the location of proposed shifts (-l flag) by estimating the BIC weights of a proposed shift at the focal node and its two daughters.

### Statistical performance of Janus

Other work by the authors showcases a range of simulated conditions under which we evaluate Janus’s performance (*30*). We previously observed that Janus is conservative, with negligible false-positive rates after removing poorly supported shifts under the BIC (i.e., with the “-u” and “-l” flags). When configured to estimate uncertainty, Janus is also robust to phylogenetic inference error (*30*). Simulations in the noted companion paper find a slightly higher rate of false negatives (0.03 to 0.05) for datasets up to 1000 bp and 250 tips (*30*). Thus, previous simulations showed that the false negative rate is low for shifts positioned randomly across simulated trees and datasets (*30*).

While our previous study showed acceptable false-positive and -negative rates for random trees simulated under a constant birth-death process (*30*), here, we evaluate performance with respect to the avian phylogeny. Specifically, the molecular phylogeny of Aves is characterized by an overall rapid and early pattern of speciation, in which many super-ordinal clades simultaneously experience early bursts of lineage accumulation (*12*, *36*–*38*). Our simulations are designed to assess how an extinction-driven bottleneck, followed by rapid cladogenesis, might affect the inference of molecular model shifts with Janus. To accomplish this, we developed a comprehensive approach starting with the phylogenetic frameworks used in this study: phylograms that reflect the variation in relative and total branch lengths across exon, intron, UTR, and mtDNA datasets. A complete pipeline (in R) for reproducing similar simulations with arbitrary topologies is provided as supplementary material, which will be helpful for other researchers undertaking similar studies. We suggest that users of Janus may use this pipeline to evaluate the performance of the method for their data.

Considering the focal phylograms, we first developed several strategies for sampling nodes to simulate nonhomogeneous substitution models. We assess three general scenarios: 1) no shifts, 2) phylogenetically independent shifts, and 3) nested shifts. The latter two scenarios are simulated according to five main parameters in a function we provide called annotate_branches (in shift_model_sims.R): a minimum clade size, a maximum clade size, the total number of shifts, a buffer constraint that maintains a minimum phylogenetic distance between selected nodes, and whether shifts should be nested or independent. For nested shifts (nested = T), the process starts with randomly sampling an initial node from a pool defined by minimum clade size. Subsequent samples are made from a pool of this node’s descendants and ancestors. Each sampled node must be a specified minimum number of steps away from any previously sampled node in terms of the number of branch paths (“buffer”). If no suitable nodes are found, the process restarts with a new initial node, repeating until the requirements are satisfied. For independent shifts (nested = F), once an initial node is selected, its lineage (descendants and ancestors) is excluded from future selections. This is repeated for a specified number of shifts, generating phylogenetically independent configurations.

The pipeline (for nested or independent shifts) starts by sampling eligible nodes based on a specified minimum clade size. In our case, we specify a minimum clade size of four (as in analyses of empirical data) and a maximum clade size set to ~3/4 of the total number of tips (~150). We also set a buffer of two. For nested shifts, this buffer setting means that sampled nodes are separated by at least one intermediate node. For independent shifts, sister clades (separated by two paths) or those more distantly related can be sampled. This set of parameters is very conservative in that it enables the pipeline to sample from a wide array of potential model parameters and shift configurations. However, our pipeline is flexible and allows a user to easily specify a more restrictive sample space if desired.

We evaluated scenarios of up to four phylogenetically independent shifts (i.e., up to five models, including the root model). In the case of nested shifts, we evaluated cases of two nested shifts (i.e., three models, including the root model). After our pipeline samples a configuration of shift nodes, they are annotated with strings of user-specified model definitions. We defined HKY models with the transition: transversion rate set to 2:1, base frequencies randomly sampled from a uniform Dirichlet distribution for each model regime (and starting root condition), and uniform site-rate patterns. For each data type (exon, intron, UTR, mtDNA) and configuration scenario (none, nested, or independent), we repeated the entire node selection process 100 times.

The outputs from our pipeline are annotated Newick strings that describe evolutionary patterns of nonhomogeneous model parameters. These strings are formatted to be parsed by the AliSim program (*101*) in IQ-Tree 2.2.2.6 (*90*) to generate simulated sequence alignments of a specified length. We used this approach to simulate alignments of 2 to 50 kbp (in the case of independent shifts or no shifts) and 2 to 100 kbp (in the case of nested shifts). In total, we generated 9200 simulated alignments to assess the performance of our approach with respect to variation in the shape of the Avian phylogeny induced by analyses of different genetic data types and shift configuration scenarios. We analyzed these simulated sequence alignments with Janus, as specified for our primary analyses; however, we omitted the -u and -l steps (for post-analysis uncertainty estimation) as well as the estimation of site-rate heterogeneity (-g, which we do not simulate) to speed up computation. These analyses required ~2 months of time distributed across ~450 AMD Threadripper CPU threads.

We estimated the average false-positive (e.g., recovery of a nonsimulated shift) and false-negative (e.g., failure to recover a simulated shift) rates for each data type (exon, intron, UTR, mtDNA) and configuration scenario. Then, we computed one-sample *t* and *Z* tests and *P* values [adjusted to control for the false discovery rate due to multiple tests (*102*)] reflecting the null hypothesis that a given average false-positive or -negative rate is not greater than zero for a given alignment length, data type, and shift configuration (nested or independent). These simulations encompass a wide array of model shift scenarios and are, therefore, intended to provide useful baseline expectations of our model and algorithm performance. Our results are reported as Supplementary text (fig. S9, A to F).

### Assessing the coincidence of substitution model shifts with the K–Pg boundary

Although we detected that only a small proportion of assessed nodes exhibit substitution model shifts (~10%), most of these are detected on nodes for which the K–Pg boundary is included within recently estimated ranges of divergence date uncertainty (*36*–*38*). In the present case, this is almost exclusively <5 to 10 Ma relative to the K–Pg boundary in the MRL3 supertree (Fig. 1 and fig. S1, A and B). To assess this hypothesis quantitatively, we modeled the Bernoulli probability of a model shift as a function of time distance to the K–Pg boundary, considering potential confounding effects of phylogenetic nonindependence, tree shape, and phylogenomic discordance.

We coded a binary dependent variable representing the presence or absence of a novel macroevolutionary regime identified by molecular model shifts and assigned 1/0 to each node using the procedure described below. This strategy is conservative, as multiple shifts in distinct genetic data types can occur along a single edge (e.g., our approach models the minimum number of implied macroevolutionary regimes). We then coded the time distance to the boundary as an independent variable, defined as the stem age for a given focal node minus 66 Ma (testing indicated a negligible impact of using stem or crown ages). These values were log-transformed after taking their absolute values, which defines our investigation in terms of proportional proximity to the K–Pg boundary. We fit models of this type with the maximum likelihood “phyloglm” approach in the phylolm R package (*103*–*105*) and compared models using the helper functions available at https://github.com/mrhelmus/phylogeny_manipulation. We additionally estimated 1000 bootstrap replicates for each data type to assess the uncertainty around model parameters.

Typically, phylogenetic regressions are applied to datasets associated with contemporary tips on a phylogeny. In our case, we assessed the probability of regime shifts tagged to internal nodes on the phylogeny (e.g., the presence or absence of a model shift in any data type). Because phylogenetic regression techniques use a variance-covariance (VCV) matrix describing shared path lengths proportional to the phylogenetic covariances of trait values (*106*), these methods are valid for phylogenies with noncontemporaneous tips. We apply this logic and assign molecular model shifts as binary states characterizing negligible-length terminals grafted to each internal node. With this “trick,” we can extend phylogenetic regression techniques to evaluate properties or states measured for internal nodes (*107*). Our overall regression strategy, therefore, relies on a modified phylogeny that includes 197 noncontemporaneous, negligible-length terminals grafted to each internal node (Supplementary R code).

Last, we investigated whether or not patterns of phylogenomic conflict hypothesized to be associated with the K–Pg boundary [e.g., (*36*, *108*)] could confound statistical associations between the probability of a novel molecular model regime and the time distance to the K–Pg boundary. This consideration serves two purposes: 1) We sought to understand how the detection of a molecular model shift may be related to potentially covarying patterns of phylogenomic discordance, which we also expect to be somewhat correlated with proximity to the K–Pg boundary (*108*), and 2) we sought to account for the fact that branch lengths estimated from concatenated datasets can contain artifacts derived from model-misspecification related to gene-tree/species tree discordance [e.g., (*109*)]. To address these possibilities, we quantified patterns of phylogenomic discordance across each data type and included a metric of discordance as an additional covariate in logistic regression models. We processed each gene tree (910 separate loci) to collapse nodes with less than 95% ultrafast-bootstrap support (*110*). We then coded each node’s percentage of discordant gene trees relative to the fixed MRL3 topology for each data type (Supplementary R Code). We applied a variance-stabilizing transformation by taking the arcsine-square root of the percentage discordance values; this transformation recognizes the biological limits on the concept of discordance while spreading out the weight of extreme values. Last, we excluded extant terminals when running logistic regression models because discordance cannot be measured for these edges. We then compared the results from alternative models considering different levels of phylogenomic discordance as a covariate (fig. S1, A to D). We depict alternative analyses considering low (mean–1 SD), mean, and high (mean + 1 SD) levels of discordance.

### Functional dimensions of sequence variation

Estimates of the optimal configuration of molecular model shifts suggested equilibrium base frequencies differed substantially across the identified regimes in each data type (Fig. 1 and fig. S7, A to D). Although our dataset was not originally conceived to examine functional characteristics of the genome (*37*), our new assembly and annotation into distinct data types (exons, introns, UTRs, and mtDNAs) presented us with the opportunity to perform a preliminary assessment of functional variation. As noted in Smith *et al.* (*30*), compositional shifts may result from nondemographic processes such as selection on codon usage for translation accuracy or even gene expression [e.g., (*111*, *112*)]. Therefore, we estimated nucleotide-based metrics to quantify the degree of codon usage bias. Further, as we generated phased haplotype data, we explored whether patterns of allelic variation in this dataset might be related to compositional shifts at the macroevolutionary scale.

For nuclear coding sequences, we evaluated three metrics of codon usage bias: synonymous codon usage order (SCUO) (*113*), effective number of codons (*N̂_c_*) (*114*), and a modified version of effective number of codons, or *N̂*′*_c_* (*115*). SCUO measures the nonrandomness in synonymous codon usage and ranges from 0 (totally random) to 1 (totally biased) and is derived from Shannon information theory (*116*). *N̂_c_* measures the effective number of codons and ranges from 20 (one codon per AA) to 61 (alternative synonymous codons equally likely). *N̂*′*_c_* additionally accounts for variation in background nucleotide composition. We, therefore, expected *N̂*′*_c_* to be the least sensitive to variation in patterns of synonymous codon usage concerning bipartitions identified partly on the basis of compositional variation. We also assessed nucleotide diversity π, as approximated by the sum of the branch lengths separating phased alleles. As with life-history traits, we log-transformed metrics of codon bias before comparative phylogenetic analysis. We estimated whether or not variation in these statistics was different across taxon partitions identified by Janus, using phylogenetic analysis of variance (ANOVA) assuming a Brownian model of trait evolution (*117*, *118*), with 10,000 simulations (*119*) to assess significance (fig. S2). Lastly, we checked to see how GC content variation at individual codon positions may contribute to macroevolutionary regimes identified by Janus in the analysis of the whole exon dataset. Taking a similar approach as above, we estimated whether or not variation in GC content was different across taxon partitions identified by Janus using phylogenetic ANOVA assuming a Brownian model of trait evolution (*117*, *118*), with 10,000 simulations (*119*) to assess significance. These supporting analyses are reported in the Supplementary text.

### Life-history and metabolism datasets

To assess how the configuration of molecular model shifts detected with Janus may be related to life-history variation, we considered how life history varies across numerous dimensions [e.g., (*11*, *120*)]. We assembled two life-history datasets to minimize the amount of missing data in each downstream analysis. The first dataset focused on quantitative life-history traits and was compiled from Bird *et al.* (*59*). These data include body mass; modeled generation length; latitude centroid; mean clutch size; annual adult survival; age at first breeding; maximum longevity; and categorically coded variables for diet, habitat, diurnality, and migratory status. We also included a metric of avian developmental mode (“ChickPC1”) that describes variation in hatchling state along an altricial to precocial spectrum (*40*). These data reflect exact species matches relative to those in the reassembled nuclear genetic dataset.

The second dataset reflects energetic constraints on life-history variation and includes basal metabolic rates (BMR) expressed in watts and associated body masses. Metabolic rates broadly scale as a ~3/4 power law function of organism mass and reflect rates of energy flow in and through organisms (*41*, *121*–*124*). Uyeda *et al.* (*29*) previously considered the hypothesis that allometric scaling parameters relating BMR and body mass have evolved across the vertebrate tree of life. We apply the same general approach to our sample of avian metabolic diversity (below). We first collected available BMR records from the AnAge senescence database Build 14 (*125*). For most of the exact species in the present dataset (and most avian species in general), conspecific BMR data have not been measured. Therefore, we conducted an extensive literature search for each avian family in the molecular dataset and filled in many missing entries by identifying phylogenetically equivalent matches (e.g., at the genus level) for which BMR and mass data were available.

Several downstream analyses required complete datasets, so we used two methods to generate unbiased estimates of missing values under a multivariate Brownian motion process (mvBM). In the case of the larger eight-dimensional breeding ecology dataset, we used Rphylopars (*126*) to fit a VCV matrix and to estimate values for missing entries. In the case of the two-dimensional metabolic scaling dataset, we used mvMORPH (*127*) to compare the fit of alternative multi-regime, mvBM models based on the model shift points identified by Janus. In the latter case, the values of the imputed data were virtually identical across alternative models (e.g., *R*^2^ > 0.98), so we selected the model with the lowest Akaike information criterion (AIC) score to use for downstream analyses.

### Analysis of life-history data

Using multiple approaches, we investigated the degree to which patterns of life-history variation reflect distinct evolutionary regimes that coincide with molecular model shifts. Several methods have been developed to automatically generate evolutionary hypotheses by identifying an optimized configuration of evolutionary models describing variation in the process of trait evolution [e.g., (*99*, *128*)], but few are expressly multivariate [e.g., (*28*, *129*)]. We investigated model heterogeneity across our high-dimensional life-history dataset with the bootstrapping approach implemented in the software ℓ1ou (*28*). ℓ1ou uses a phylogenetic lasso method to identify points on a phylogeny where a trait’s optimum value θ(*t*) has shifted, assuming α (the “pull” toward the optimum or adaptation rate) and σ^2^ (the Brownian diffusion rate parameter) are fixed across the tree. The ℓ1ou approach is extended to multiple traits by assuming that traits shift their optimum simultaneously and in the same location on the tree (*28*).

Conveniently, ℓ1ou allows the researcher to specify a set of candidate edges for the lasso approach to consider for shifts in θ(*t*). This attribute allows us to articulate the specific models we want to compare. We ran ℓ1ou with a constrained set of candidate edges reflecting the 12 candidate shift edges identified across analyses of different molecular data types (Fig. 1 and table S1). Thus, for a given ℓ1ou analysis of this type, ℓ1ou can infer 0 to 12 shifts in θ(*t*). We repeated this procedure with the AICc and pBIC information criteria, as recommended by Khabbazian *et al.* (*28*), and used 100 bootstrap replicates to assess the positive detection rate for each candidate edge. In these analyses, our intention is not to identify every case where a life-history shift may have occurred across avian phylogeny; our goal is to assess how much statistical support exists for shifts in life-history trait optima that coincide with shifts identified in our analysis of molecular data. We validated these results by comparing them to a null distribution of shift detections reflecting the false-positive rate under multivariate Brownian motion [e.g., without shifts in θ(*t*)]. Using the eight-dimensional VCV matrix estimated by RPhylopars (*126*), we simulated 500 null datasets using the function simRatematrix in the R package ratematrix (*130*). We then analyzed each simulated dataset with ℓ1ou as previously specified. For each candidate edge, we used Fisher’s exact test (*131*) to assess whether the frequency of positive shift detections in the empirical dataset was significantly greater (one-tailed *P* value = 0.05) than the null false-positive rate observed across simulated datasets. Tables of *P* values and odds ratios are reported as Supplementary material (fig. S4A and table S2). Last, we investigated which life-history traits were most closely associated with molecular model shifts using a machine-learning approach implemented in the tidymodels framework (below) (*132*).

We performed additional, unconstrained analyses with ℓ1ou to evaluate how well the ℓ1ou framework detected shifts in θ(*t*) that correspond with those identified by Janus. Taking our eight-dimensional dataset, we ran ℓ1ou under default search parameters with three available information criteria (AICc, BIC, and pBIC). These additional tests were set to consider the same candidate nodes in analyses of molecular data (i.e., all edges with ≥4 descendant lineages). We visually assessed the temporal sequence of resultant ℓ1ou shifts with two approaches. First, we visualized the relative density of ℓ1ou shifts through time, using the generic Kernel density estimator built into R (*133*), to examine whether ℓ1ou detected any shifts far from the K–Pg boundary. Next, we wrote new R functions to identify which set of reference shifts (identified by ℓ1ou) was closest to a given target shift (identified by Janus) and to return their path distance (in nodes). Taking these path distances as discrete data for each target shift (i.e., number of nodes), we visualized density plots using the estimator for discrete data in the R package kde1d (*134*). These supporting analyses are reported as Supplementary text (fig. S4B).

### Assessing feature importance with a random forest classifier

To identify which, if any, life-history traits may be good predictors of molecular model shifts, we used an approach from the field of supervised machine learning known as random forests (*135*, *136*). The random forests approach generates a classification model based on a population of decision trees (*137*) and can naturally assess the relative importance of different features (*138*). Here, we focus on the prediction of taxon partitions (groups of terminals) identified in analyses of exon data, although alternative analyses of taxon partitions identified in other data types generated similar results (not shown). Although it may be possible to directly incorporate aspects of phylogenetic distance into these analyses (*138*, *139*), nonparametric machine learning methods like random forests make no assumptions about the distribution of the underlying data and can handle skewed or multimodal data as well as categorical data; thus, accounting for phylogenetic nonindependence in the data is not expected in the same way as when conducting, for example, a generalized least squares (GLS) analysis. Nonetheless, we checked for the impact of phylogenetic signal by running a parallel analysis of phylogenetic residuals, assuming a Brownian motion model (Y~1); ultimately, this did not affect our ranking of feature importance and is not discussed further.

First, we split our life-history data into training and test datasets with a 70/30 split, accounting for stratified sampling. We then used tidymodels (*132*) to build a recipe for data preprocessing, specifying several steps: 1) removing any variables correlated with others at a Pearson correlation coefficient > 0.95, 2) normalizing (centering and scaling), 3) creating dummy variables for categorical variables with one hot encoding, and 4) generating synthetic positive instances using ADASYN algorithm (*140*) to increase the sample size of small groups to at least 50% of the size of the largest group (setting the number of neighbors to 2).

Next, we specified the structure of the model, including hyperparameters “mtry” (number of features to sample, set to tune automatically) and “min_n” (minimum number of data points in a node to allow further splitting, set to tune automatically). We set the number of trees in a forest to 1000 and specified the “randomForest” engine (*141*). To tune the hyperparameters, we used k-fold cross-validation with 10 folds repeated 10 times. We selected the best model from the hyperparameter tuning based on the AUC using the estimator from Hand and Till (*142*) and fit it to the training set. The AUC can be interpreted as the probability that a randomly chosen positive example is ranked above a randomly chosen negative example and ranges from 0 to 1, with values closer to 1 reflecting better model performance. Last, we estimated permutation-based variable importance (*143*) using the VIP R package (*144*) with 500 simulations (Fig. 2, left). See supplementary R script RandomForest_var_imp.R for details.

To further investigate these results, we fit fixed shift OUM [shifting θ(*t*), with fixed α and σ^2^; equivalent to that used by ℓ1ou] models using OUwie (*27*) to the two most important features identified by the random forest classifier. This model is a much better fit to the data than a single-peak OU model (e.g., ΔAIC_mass_ = 67.5). We used 100 parametric bootstrap replicates to estimate model parameter uncertainty (Fig. 2, right). We also simulated 1000 datasets under each fitted OUM model to visualize diagnostic distributions of expected trait values at the present (e.g., tip data simulated under the fitted model; Fig. 2, right). These models, therefore, indicate the expected shifts in trait optima θ(*t*) that coincide with bipartitions identified in the analyses of molecular data.

### Analysis of metabolic rate data

To assess whether molecular model shifts may be associated with shifts in patterns of metabolic scaling, we assessed support for coincident shifts in patterns of metabolic allometry. We used the Bayesian phylogenetic framework implemented in the R package bayou 2.0 (*29*, *128*). bayou applies a reversible jump Markov chain Monte Carlo approach to detect the magnitude, number, and phylogenetic position of model shifts. Using bayou, we implemented an allometric regression model that relates BMR and body mass logarithmically and for which slope β_mass_ and intercept β_0_ evolve under a multi-regime OU process. Here, model shifts reflect shifts in the optimum of the evolutionary allometry between BMR and body mass.

Using the rjMCMC approach in bayou, we estimated the posterior probability of an allometric shift occurring along the 12 candidate edges identified by Janus. Under a Poisson prior, we specified the mean number of shifts across the phylogeny reflecting 2% of the total edges in the tree (λ = 8) with equal probability. In this context, maximal posterior probability indicates an increase over the prior probability by ~50%. We ran each analysis across three replicate chains for 10 million iterations, sampling every 1000 iterations. Given that we did not have consistent estimates of measurement error, we followed the approach of Uyeda *et al.* (*29*) and explored alternative analyses assuming an SE of 0.1 or 0.01 for BMR and recovered a negligible impact (not shown).

Our priors for α, σ*^2^*, β_mass_, β_0_ reflect half-Cauchy or Gaussian expectations:

$$\alpha ∼\text{half}–\text{Cauchy}\;\left(\text{scale}=0.1\right);{\mathrm{myr}}^{-1}$$

$$\sigma^{2}∼\text{half}–\text{Cauchy}\;\left(\text{scale}=0.1\right);\frac{\text{ln}\left(\mathrm{BMR}\;\text{watts}\right)}{\mathrm{myr}}\;$$

$$\beta_{\text{mass}}∼N\;\left(\mu =0.7,\sigma =0.1\right);\frac{\text{ln}\left(\mathrm{BMR}\;\text{watts}\right)}{\text{ln}\left(\text{body mass}\;g\right)}$$

$$\text{ln}\left(\beta_{0}\right)∼N\;\left(\mu =-3.5,\sigma =1.75\right);\text{ln}\left(\mathrm{BMR}\;\text{watts}\right)$$

Replicate analyses with rjMCMC identified numerous shifts in the slope and intercept at a posterior probability cutoff of 0.1 after discarding the first 40% of samples as burn-in. Following rjMCMC runs, we reestimated model parameters on a fixed configuration model reflecting all molecular shifts identified with Janus (Fig. 3). We assessed model convergence by examining Gelman and Rubin’s R statistic (*145*, *146*) and effective sample sizes across chains and parameters (see table S1 and the BMR directory on the author’s GitHub repository; https://github.com/jakeberv/avian_molecular_shifts/tree/main/BMR). Last, we visualized how the estimated allometric slope and intercept parameters scale with the body masses of species within the specified shift regimes (fig. S10).

### Linkages among GC content, effective population size (N_e_), and body mass

We checked to see whether the average clade body mass within groups identified by Janus corresponded to average GC content or estimated equilibrium GC content (fig. S8). We find strong negative relationships consistent with a mechanism of GC-biased gene conversion in each case, assuming that N_e_ is broadly and negatively correlated with body mass. The relationship between N_e_ and body mass is generally accepted to result from the relationship between environmental carrying capacity and body mass (e.g., there are fewer ostriches than sparrows) (*18*, *19*, *147*–*149*). Here, we share the results for exons and introns, which have a sufficient number of identified regimes to perform this check, although the patterns were similar for UTRs and mtDNA (fig. S8). These results recapitulate the patterns shown in Fig. 1 (bottom), indicating a more significant deviation between empirical and estimated equilibrium base frequencies for coding than noncoding sequences. We speculate why these deviations may be more pronounced for exons within the manuscript text (e.g., functional constraints, codon usage bias, recombination, selection, or DNA polymerase function). These patterns are generally consistent with those previously reported in the referenced literature (*21*, *64*) and support GC-biased gene conversion as a mechanism driving the patterns we detect (e.g., *R*^2^ values >0.5).

## Supplementary Materials

### This PDF file includes:

Supplementary Text Figs. S1 to S10 Tables S1 and S2 Legends for tables S3 and S4 References
