# Supplementary material for: Genome and life-history evolution link bird diversification to the end-Cretaceous mass extinction
Source: Sci Adv. 2024 Jul 31;10(31):eadp0114. doi: 10.1126/sciadv.adp0114 (PMC11290531; doi:10.1126/sciadv.adp0114)
Supplement: Supplementary file 1 — Supplementary Text Figs. S1 to S10 Tables S1 and S2 Legends for tables S3 and S4 References [file sciadv.adp0114_sm.pdf]

Science Advances
AAAS

Supplementary Materials for

Genome and life-history evolution link bird diversification to the end-Cretaceous mass extinction

Jacob S. Berv *et al.*

Corresponding author: Jacob S. Berv,jberv@umich.edu, jacob.berv@gmail.com

Sci. Adv. **10**, eadp0114 (2024)
DOI: 10.1126/sciadv.adp0114

The PDF file includes:

Supplementary Text
Figs. S1 to S10
Tables S1 and S2
Legends for tables S3 and S4
References

Other Supplementary Material for this manuscript includes the following:

Tables S3 and S4

Supplementary Text

Molecular and life-history datasets

Our final combined nuclear genetic dataset is a matrix of 400 rows (two alleles per taxon for each locus) and 682,178 aligned base pairs. These data reflect an effective increase in called bases by a factor of ~3.45 and an increase in alignment length by a factor of 1.73 compared with the original dataset from Prum *et al.* 2015 (*37*), accompanied by increases in the percentage of undetermined and gap characters of less than 10% overall. Only ~14% of the characters in the combined dataset are gaps "-", and ~8% are undetermined characters. The final combined mitochondrial dataset encompasses all 15 targeted regions (13 proteins and 2 rRNAs) and 200 taxa with an average alignment length of 13,769 base pairs (Table S3). Both life-history datasets reflect the phylogenetic sample of avian lineages in Prum *et al*. 2015 (*37*). For the first dataset, we assembled data for body mass (0% missing), modeled generation length (0% missing), latitude centroid (0% missing), mean clutch size (~13.6% missing), annual adult survival (77% missing), age at first breeding (58% missing), maximum known longevity (54.5% missing), and developmental mode ("ChickPC1" – 0% missing). Categorical variables have no missing data (Fig. 2, left). For the second dataset highlighting avian metabolism, our procedure to identify phylogenetically equivalent swaps generated ~70% coverage for BMR and 100% for body mass (Table S4a).

Molecular dataset details

The percentage of undetermined 'N' characters was relatively low in exons (~1.3%) but substantially higher in introns (~24%) and UTRs (~18%). Our final filtered dataset comprised 384 exons (average ~1,198bp), 365 introns (average ~404bp), 147 UTRs (average ~453bp), and 14 loci which were noncoding but did not clearly map to a non-coding genic region (average ~578bp). The reprocessed dataset also reflects an increase in the number of parsimony informative sites by a factor of 2.18 for the phased alignment (~1.8 for a single allele) relative to the Prum *et al.* 2015 (*37*) dataset. These encouraging results imply that many previously generated phylogenomics datasets may be effectively reprocessed with updated approaches to generate significantly larger and well-annotated resources for studying phylogeny and molecular evolution. Due to computational bottlenecks, our molecular model shift analyses were conducted on sub-sampled sequence datasets representing one sampled allele from each species (we provide the re-assembled datasets as supplementary data). Lastly, the final combined mitochondrial dataset encompassed all 15 targeted regions (13 proteins and 2 rRNAs) and covered 200 taxa with an alignment length of 13,769 base pairs (Table S3). These data also have a low percentage of missing or undetermined characters (12.8%).

Assessing the coincidence of substitution model shifts with the K–Pg boundary

Phylogenetic logistic regression models show a rapid increase in the probability of a molecular model shift as the time distance to the boundary decreases (Fig. S1a-d). In all models (except those representing mtDNA), time distance to the boundary is a significant predictor of model shift probability ($p << 0.05$). Considering low (Fig. S1b), mean (Fig. S1c), or high (Fig. S1d) values of phylogenomic discordance has a limited effect on the predicted probability of model shifts. Considering the predicted probability of model shifts across the "merged" nuclear data signal at the time of the oldest shifts in the dataset (marked with leader lines; black curves in Fig. S1), we find a weak inverse relationship between low, mean, and high values of discordance, and model shift probability [63%, 59%, 55%]. This implies a very weak confounding effect, with higher levels of discordance leading to a slightly weaker probability of substitution model shifts. These trends are shared with patterns discordance and model shifts in exon and UTR datasets [42%, 32%, 24%, and 44%, 37%, 30%, respectively]. The signal of shifts in introns, however, shows the opposite trend, with patterns of low, mean, and high discordance leading to a linear increase in shift probability [19%, 26%, and 34%, respectively], implying that the dynamics of model shifts in introns may be different from those in other nuclear data types. In any case, the bootstrapped confidence intervals for different levels of discordance across introns largely overlap, challenging interpretations.

In sum, and in general, considering phylogenomic discordance in these models does not reject an inference of an association between time distance to the K–Pg boundary and an increased probability of substitution model shifts. Phylogenomic discordance itself is not a significant covariate in any individual pGLM model, and models that exclude discordance entirely are weakly to moderately preferred under AICc (~ 1.5 AICc unit for the "aggregate" model, < 1 AICc units for exons, ~5 AICc units for introns, and ~1.1 AICc units for UTRs). Notably, while there is some collinearity between time distance to the boundary and discordance, the association is only strong in non-phylogenetic regression models (not shown but see Supplementary R code). Thus, models that account for phylogeny may also largely account for collinearity between discordance and time distance to the boundary.

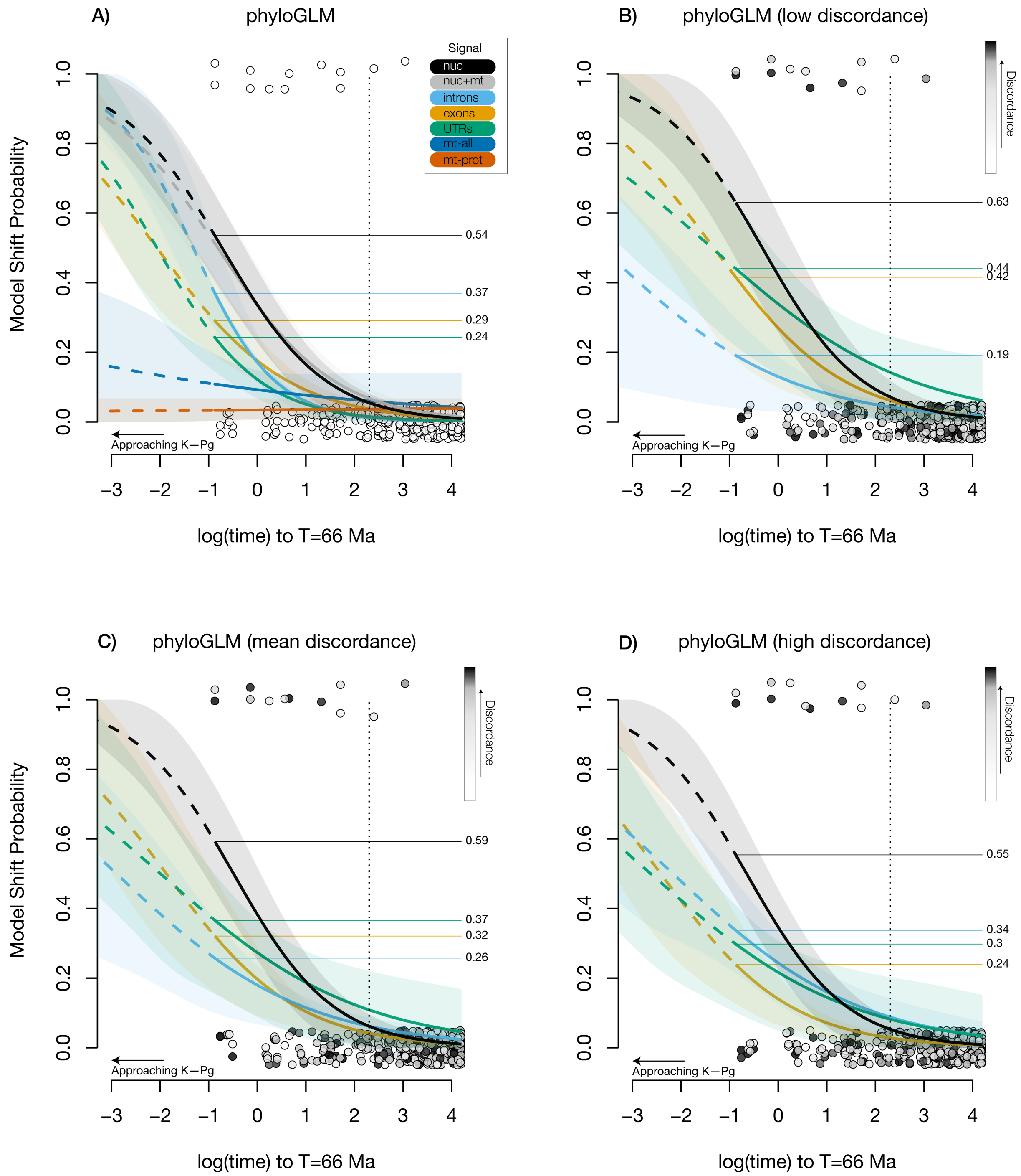
A)
phyloGLM
Signal
nuc
nuc+mt
introns
exons
UTRs
mt-all
mt-prot
0.54
0.37
0.29
0.24
Approaching K−Pg
Model Shift Probability
log(time) to T=66 Ma
B)
phyloGLM (low discordance)
Discordance
0.63
0.44
0.42
0.19
C)
phyloGLM (mean discordance)
0.59
0.37
0.32
0.26
D)
phyloGLM (high discordance)
0.55
0.34
0.3
0.24

Fig. S1. Model shift probability as a function of time distance to the K–Pg boundary. Binomial probability of novel molecular model regimes as a function of time distance to the K–Pg boundary (e.g., time distance approaches zero, going from right to left, log scale). Logistic regression curves and associated 70% highest density intervals from bootstrap replicates are depicted over the jittered empirical probability distribution for individual nodes (circular markers). Model projections outside the range of the observed data are shown as dashed curves. In panel A, the probability of a model shift in the aggregate signal from nuclear genetic data (black curve) increases to ~54% at the time of the oldest model shift. The probabilities of shifts across each nuclear genetic data type are somewhat lower (~26%-37%). In contrast, the probability of a shift in mtDNA (all data, or proteins only) is not associated with time distance to the boundary (flat curves). This pattern for mtDNA is consistent with the overall low number of shifts inferred across mtDNA genomes ($n < 3$). Panels B-D depict logistic regression curves from models that include phylogenomic discordance as a covariate and show patterns of phylogenomic discordance with a white-to-black color ramp. Discordance is not detected as a significant covariate in any model, and models with discordance have higher AICc values, suggesting they are not a better fit to the data. Nevertheless, comparing model predictions with low, mean, and high discordance values shows a very weak confounding effect, with higher levels of discordance leading to a weaker predicted probability of model shifts, except for introns (see supplemental text).

Functional dimensions of sequence variation

We found no statistical evidence that patterns of nucleotide diversity are different across molecular model shift regimes after accounting for non-independence due to phylogeny ($F = 0.933$ $p = 0.98$), though we emphasize that this is an exploratory analysis in the absence of population-level sampling at the species level. Next, we found that patterns of synonymous codon usage may be significantly different across model regimes in the omnibus ANOVA, and for many cases of post-hoc comparison after correction for multiple hypothesis tests (SCUO; F: 17.6, $p = 0.005$, $\hat{N}_c$; F: 17.23, $p = 0.005$, $\hat{N}'_c$ F: 27.23, $p = 0.0003$). These results are depicted in Fig. S2. Notably, the $\hat{N}'_c$ metric appears to be the most sensitive to patterns of variation in codon usage, identifying many cases in which post-hoc pairwise comparisons are statistically different. For example, with $\hat{N}'_c$ the unnamed sister clade to Tinamiformes (a clade uniting Rheiformes, Casuariiformes, and Apterygiformes) appears to have the strongest signal of differentiation, with 5/7 comparisons implying significantly different patterns of codon usage (Fig. S2), sans the groups Passeri or Tinamiformes). In sum, for all eight groups identified by *Janus* as occupying distinct molecular model regimes in exons, we find evidence of significant differences in patterns of codon usage in comparison to at least one other candidate group. Although it was not our focus here, these results strongly imply that identifying compositional shifts with methods like *Janus* may be fruitful in generating hypotheses about functional variation in coding sequences.

Interestingly, we find that, after accounting for phylogeny, variation in empirical GC content across all codon positions significantly contributes to taxon partitions identified by *Janus*. Considering the simple formula: Percentage of Variance Explained by x = (Sum Sq for x / Total Sum of Squares) * 100, we find variation in codon position 1 (47%) contributing similarly to variation in codon position 3 (52%). Codon position 2 contributes a lesser, but not insignificant amount (28%). For positions 1 and 3, the omnibus test reveals a statistically significant result after accounting for phylogeny ($p < 0.001$). In contrast, the lower amount of explainable variance is reflected in a higher p-value for codon position 2 ($p = 0.07$). In sum, an examination of the empirical data was consistent with our previous understanding of sequence variation as well as an important role for all codon positions within the coding sequence in the present context. Notably, these patterns (see supplementary R code) are not consistent with an outsized role of the third codon position in driving our results – however, they are consistent with patterns of codon usage as an additional, linked dimension of variation.

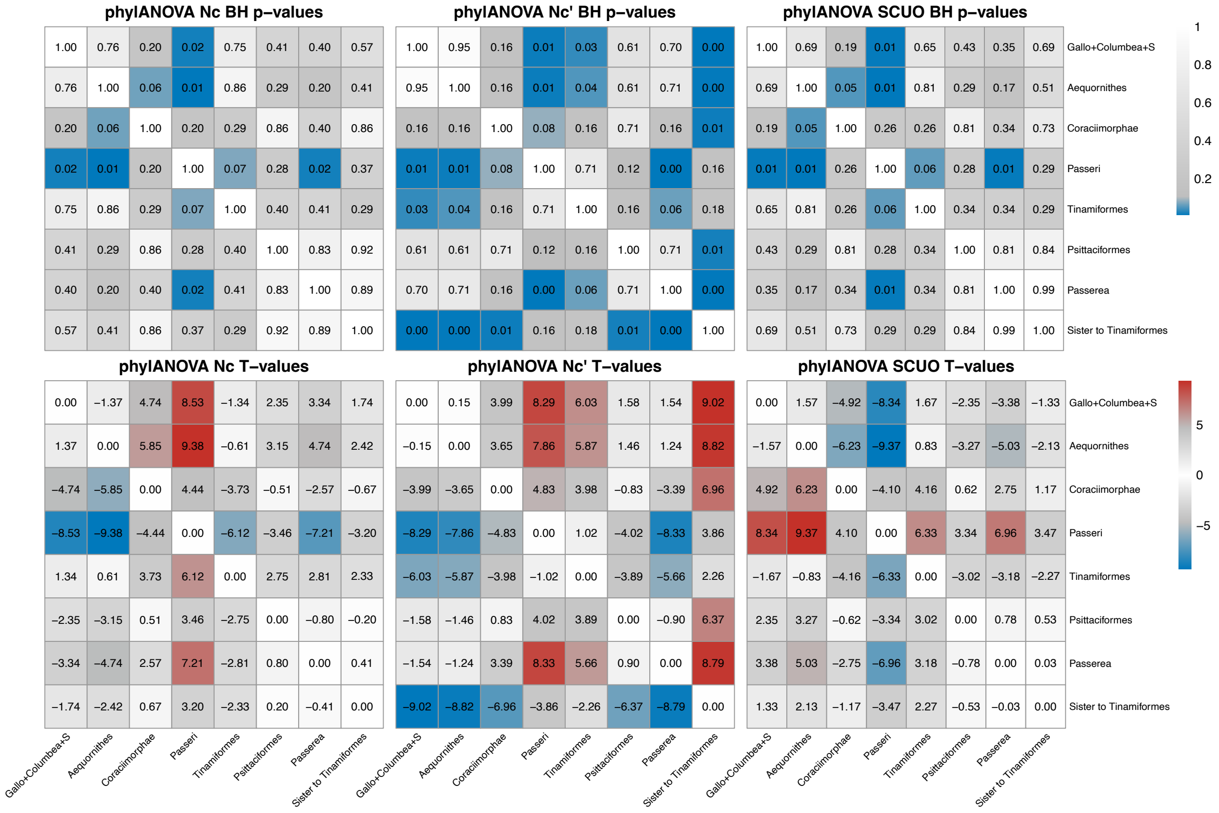

Fig. S2. Synonymous codon usage patterns are associated with molecular model shifts. For exon data, we evaluated whether patterns of synonymous codon usage differed across phylogenetic regimes identified in the analysis of molecular model shifts. In the top row, we depict matrices of Benjamini-Hochberg-adjusted p-values (*102*) for post-hoc pairwise comparisons from phylogenetic ANOVA. In the bottom row, we depict T-values for each respective test. See the supplementary text for additional details.

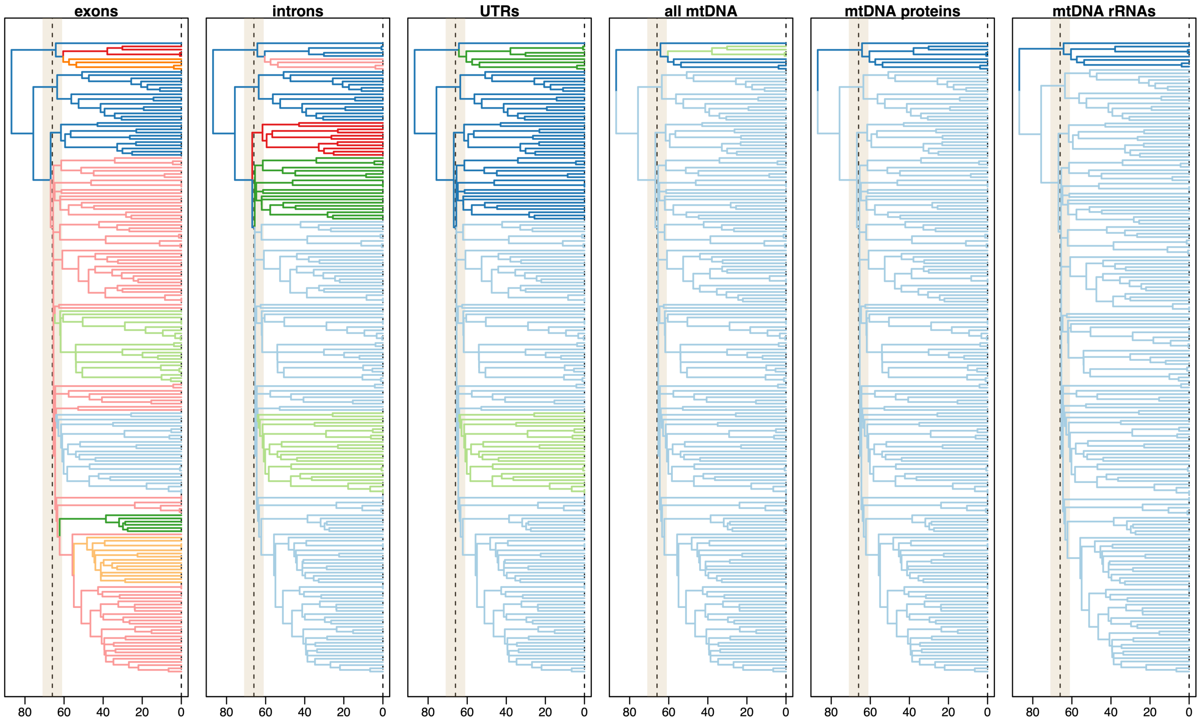

Fig. S3. Patterns of molecular model shifts for each evaluated data type. Shifts in branch color indicate model shifts. Fig. 1 contains this information in aggregate. Model shifts are most common in exon data, followed by introns, UTRS, and mtDNAs. We note that two model shifts are detected in the analysis of the whole mtDNA dataset (Neognathae and Tinamiformes), whereas analysis of mtDNA proteins or rRNAS separately identifies the same model shift (Neognathae only). We interpret this difference to reflect increased power from the combined mtDNA dataset. See Fig. S7a-d for an expanded view.

Assessing the coincidence of molecular shifts and shifts in θ(t)

As described in the materials and methods, we assessed statistical support for the hypothesis that shifts in θ(t) may have co-occurred with molecular model shifts using a bootstrapping approach implemented in ℓ1ou (*28*). Under models relying on the AICc criterion, median support was 87.2% [0.59-0.91] for shifts across the eight-dimensional dataset (pie charts in Fig. 1, Fig. S2). Considering the more conservative pBIC criterion, median support was modestly lower: 76.2% [0-97.3]. For the latter case, three candidate edges received no support relative to the false positive rate: the edge leading to Oscine Passeriformes and the two edges reflecting the stem of Otidae or its unnamed sister clade. Notably, these latter two edges reflect the two deepest splits in Passerea (*36*), while the edge leading to the MRCA of Passerea receives the strongest relative support under pBIC (97.3%). Applying Fisher's exact test to these inferences supports all cases except those noted above, for which a test of equality in proportion is not rejected at $p = 0.05$. All other cases are supported at $p < 10^{-4}$ under both AICc and pBIC except for two cases: Notopalaeognathae (AICc: $p = 0.238$, OR = 1.49; pBIC: $p = 0.04$, OR = 2.1), and the sister clade to Tinamiformes (AICc: $p = 0.015$, OR = 2.19, pBIC: $p < 10^{-4}$, OR = 4.7) (Fig. S4a, Table S2).

Additionally, unconstrained analyses with ℓ1ou detected 5/12 perfect matches (asterisk inside a circle, Fig. S4b), compared to shifts identified by *Janus*. All shifts identified by *Janus* are ~ 1-2 edges away from a shift identified by ℓ1ou in at least one analysis (e.g., pBIC). Thus, we show a tight congruence between patterns identified in molecular data and those identified in life-history data, even when analysis of the latter is not informed by molecular data. We find additional shifts when using ℓ1ou in an unconstrained fashion. However, these shifts are mostly restricted to a temporal interval closely associated with the K–Pg boundary (density plots in S4b). Analyses using the pBIC criterion–the criterion recommended by the authors of ℓ1ou (*28*)–show the closest correspondence with *Janus*. The other shifts identified with ℓ1ou are worthy of further examination—however, we omit further discussion of them here as they are of limited relevance in the context of the present manuscript.

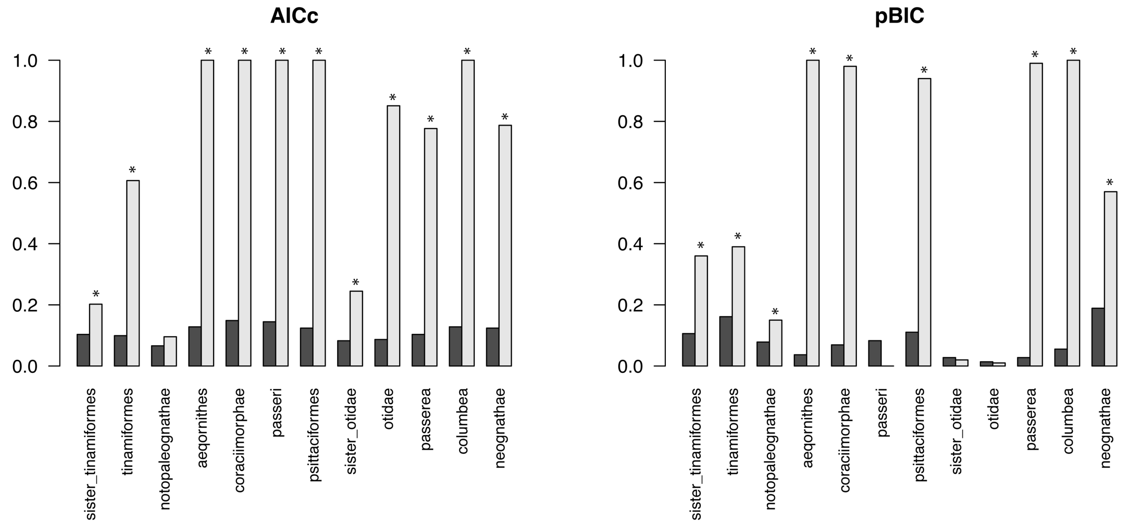

Fig. S4a. Relative support for shifts in trait optima θ(t). For each criterion (AICc or pBIC), we depict the frequency of empirical positive shift detections from ℓ1ou bootstrapping (light gray) relative to the frequency of false positive detections (dark gray) generated with a dataset simulated under multivariate Brownian motion (e.g., no shifts in θ). Cases in which the empirical positive detection rate is significantly greater than the false positive rate (Fisher's exact test) at p = 0.05 are marked with an asterisk (see materials and methods and Table S2).

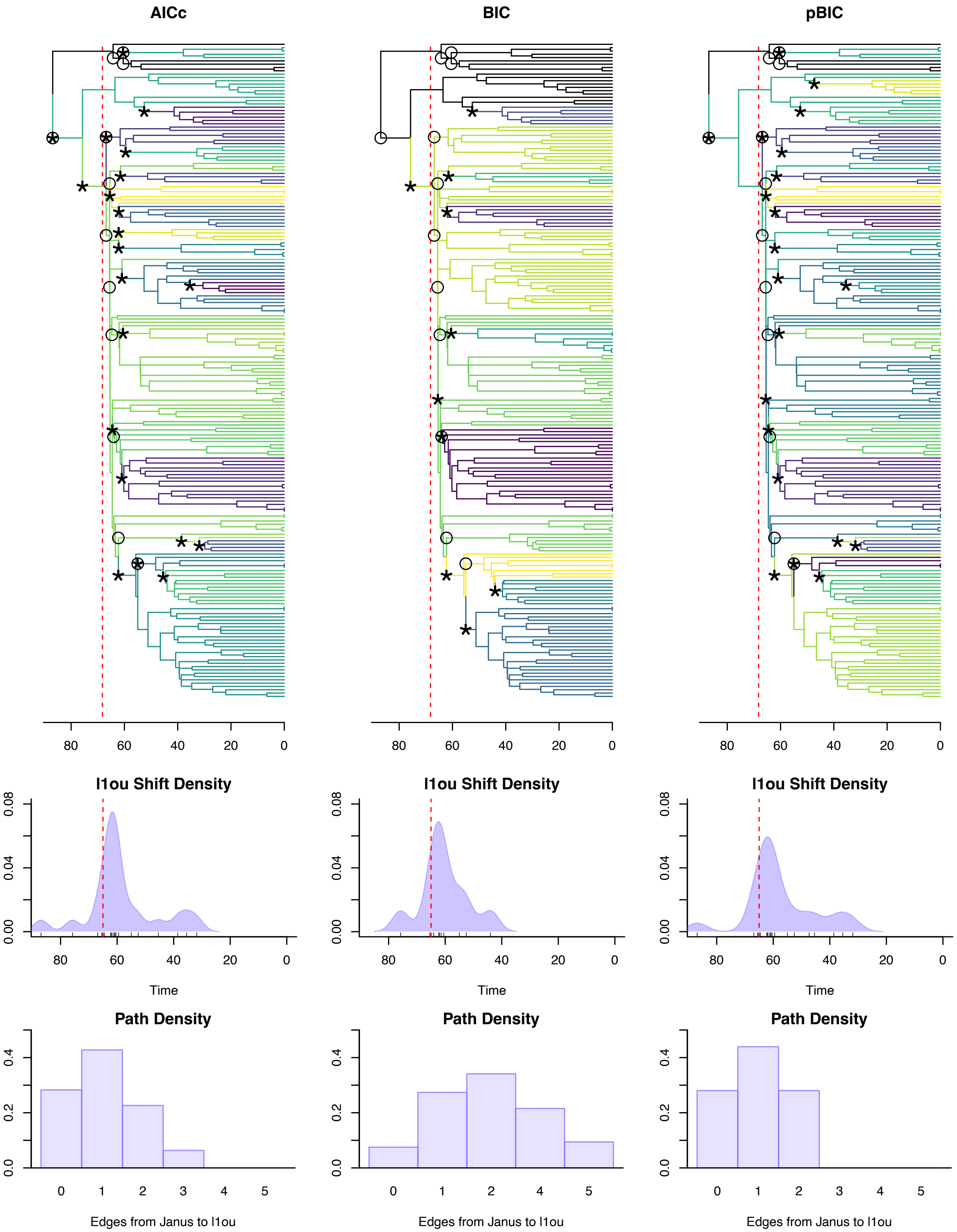

AICc
BIC
pBIC
80
60
40
20
0
l1ou Shift Density
0.08
0.04
0.00
Time
Path Density
0.4
0.2
0.0
0
1
2
3
4
5
Edges from Janus to l1ou

Fig. S4b. Unconstrained ℓ1ou analyses. This figure shows results evaluating ℓ1ou and our eight-dimensional life-history dataset across three information criteria (AICc, BIC, and pBIC). In the top row, circles mark edges identified across all analyses using *Janus*, indicating candidate molecular model shift points. Asterisks and phylogeny edge colors indicate shift points identified by ℓ1ou, representing shifts in the multivariate phenotypic optimum $\theta(t)$. The second row of the figure shows the temporal sequence of ℓ1ou shifts, indicated by relative density. These plots show that few shifts are detected far from the K–Pg boundary in unconstrained analyses in ℓ1ou. The third row summarizes how many edges separate a given shift point *Janus* identified to the closest shift identified by ℓ1ou. In addition to 5/12 perfect matches (asterisk inside a circle), all shifts identified by *Janus* are < 1-2 edges away from a shift identified by ℓ1ou in at least one analysis (e.g., pBIC).

Supplemental models of life-history traits

In the main text, we show visualizations of OUM models in which trait optima θ(t) can shift across specified regimes (Fig. 2, right panel); in this case, macroevolutionary regimes are defined *a priori* at molecular model shift points. These results stem from an analysis of nuclear genetic data, emphasizing molecular changes associated with the radiation of Neoaves. Indeed, for most cases within this superordinal clade, we observe decreases in the estimates for θ(t) associated with molecular model shifts relative to the ancestral regime θ_{anc}. Based on our random forests classifier, we visualized two features with the highest relative variable importance scores ("ChickPC1" and "adult body mass," Fig. 2, Right). Notably, this set of taxon partitions from *Janus* includes a plesiomorphic group, uniting our single lineage of *Struthio* with the clade Galloanserae on the same molecular regime (e.g., Supplemental Fig. 7a). To explore these patterns further, we fit additional OUM models, placing *Struthio* on a separate regime along with the tree's root. This configuration reflects the aggregate signal of taxon partitions identified across nuclear and mitochondrial datasets (Fig. 1, e.g., adding the shift detected in mitochondrial data for Neognathae). By placing the large-bodied *Struthio* lineage on a separate regime, we can estimate how its closest sister clades may vary in θ(t) relative to the ancestral regime without being potentially confounded by the inclusion of Galloanseres. For these analyses, we re-estimated OUM models for all numerical life-history traits (Fig. S6, also see Fig. S5).

Lastly, Uyeda *et al.* (*151*) describes how the analysis of principal components can, under some circumstances, lead to misleading phylogenetic inferences. Specifically, Uyeda *et al.* (*151*) describes a phenomenon by which ordination partitions the variance of a multivariate trait such that the major component axes are biased toward "early burst" patterns. As one of the features we examine is a principal component of multivariate developmental mode (*40*), we checked for this bias by fitting univariate EB, OU, and BM models to each of the eight examined life-history traits and then comparing model fit with AIC weights. We performed these checks using the *fitContinuous* function in (*117*) and further validated the results with *OUwie* (*27*).

Placing *Struthio*+root on a separate macroevolutionary regime revealed more complex dynamics within the Palaeognathae (Fig. S6). The *Struthio*+root regime is detected to have a much larger θ(t), magnifying the apparent shift in life-history trait θ(t) for all other taxon partitions identified by molecular model shift analysis. In this configuration, all candidate regimes identified by *Janus* show reduced θ(t) for ChickPC1 or adult body mass relative to the ancestral regime. We observe similar patterns for clutch size, and a somewhat weaker signal for generation length (Fig. S5, S6). These analyses corroborate patterns revealed by random forest variable importance analysis (materials and methods), which identified "ChickPC1" and adult body mass as the features that best predict molecular substitution model shifts. Given the challenges in accurately estimating evolutionary parameters like θ(t) from small sample sizes, we present these additional analyses as supporting text. Nonetheless, all estimates are consistent with patterns described in the main text. Further, checks for a bias toward early burst patterns in the data did not reveal any life-history traits for which "EB" was selected as the preferred model. "OU" models carried nearly 100% of the model weight in all cases (OU > BM > EB in all cases; see Supplementary R code). Thus, at the scale of the examined phylogeny, we do not see evidence that ordination (in the case of developmental mode data, *40*) has biased these data toward an early burst pattern.

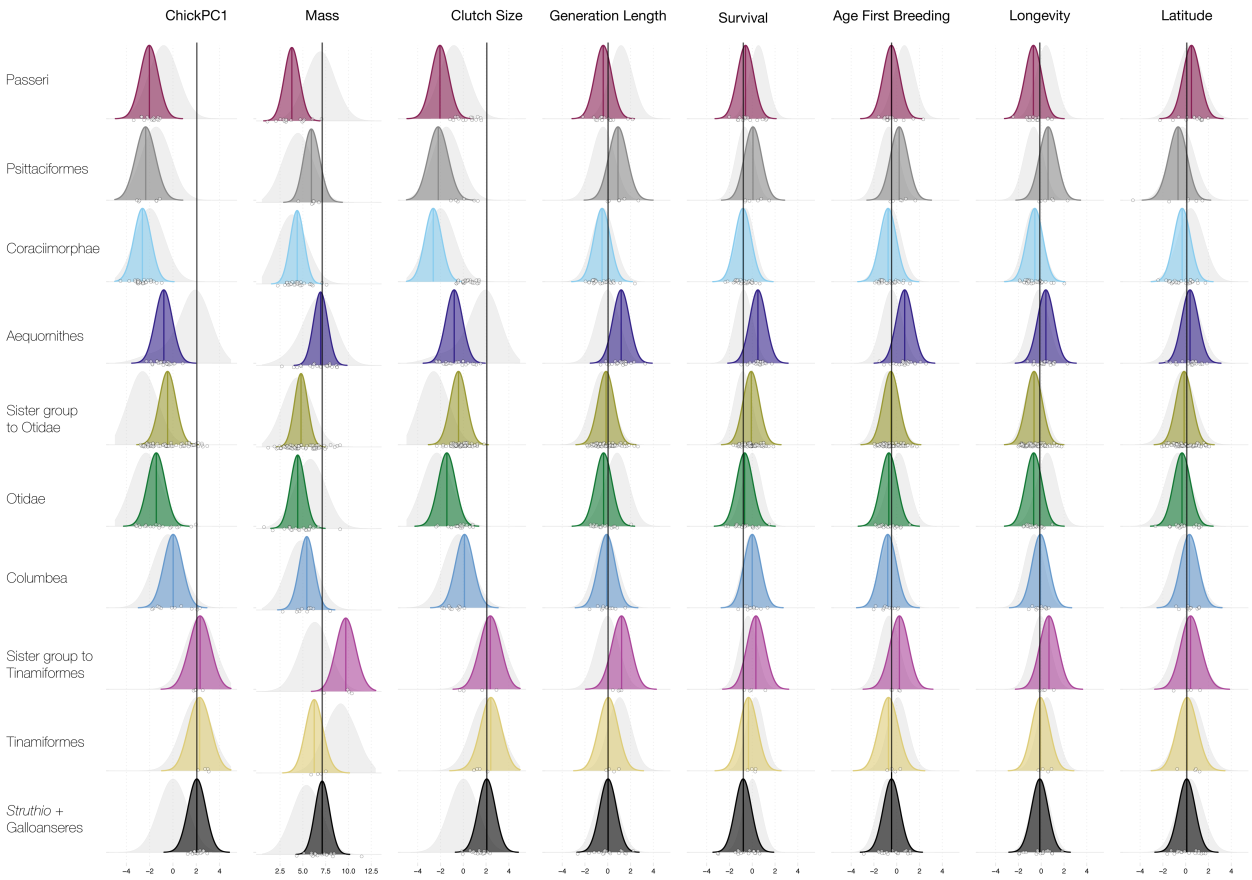

Fig. S5. Complete set of θ(t) shift models (nuclear data configuration). We estimated shifts in θ(t) under an OUM model in OUwie (*27*). We describe the results for ChickPC1 and adult body mass in the main text, as these two features are identified as being most important with a random forest classifier (See main text and supplementary text). Here, we show the complete set of models for each of the eight continuous life-history traits. Colored distributions reflect parametric bootstrap estimates of θ(t), while grayed-out background distributions reflect a summary of 10,000 simulations of tip values from the model (e.g., time = 0). Notably, analysis of clutch size reveals similar patterns to those observed for ChickPC1 and adult body mass (namely, a reduction in the estimated values for θ(t) for the ancestral regime for many Neoavian clades). Although some fluctuations are observed for other traits, none are as pronounced as for ChickPC1 and adult body mass.

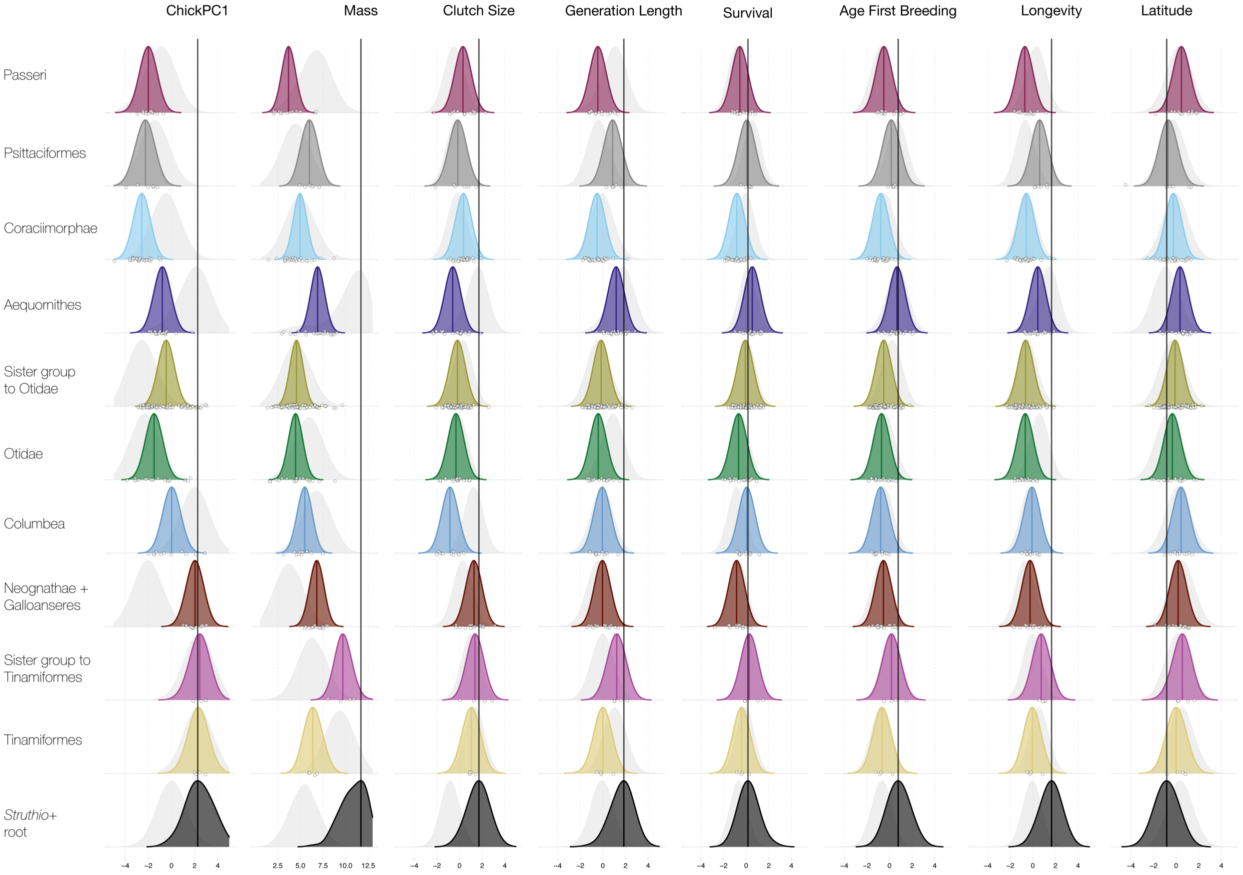

Fig. S6. Complete set of θ(t) shift models (nuc+mt data configuration). We estimated shifts in θ(t) under an OUM model in OUwie (*27*). We present ChickPC1 and adult body mass results in the main text, as these two features are identified as most important in a random forest classifier (See main text and supplementary text). Colored distributions reflect parametric bootstrap estimates of θ(t), while grayed-out background distributions reflect a summary of 10,000 simulations of tip values from the model (e.g., time = 0). Here, we show the complete set of models for each of eight continuous life-history traits– with an essential difference from the results presented in Fig. 2 and Fig. S5. Here, an additional partition is included, reflecting a unique molecular model shift identified in the analysis of mtDNA (Neognathae). This has the effect of placing *Struthio* on a regime separated from Galloanseres (e.g., Fig. 2, Fig. S5). The Galloanseres are thus united with a regime including the Neognathae MRCA. Shift patterns for ChickPC1 and clutch size θ(t) are essentially identical to those presented in previous analyses – however, investigation of body mass reveals more complex dynamics within the Palaeognathae. The *Struthio* regime contains the tree's root and is naturally estimated to have a much larger θ(t). This, in turn, induces an apparent decrease in θ(t) for body mass in Tinamiformes and more substantial decreases in θ(t) for all other cases. As noted in the supplementary results, these patterns suggest that *all molecular model shifts* (either nuclear or mtDNA) indicate derived decreases in body mass or ChickPC1 θ(t). Similar fluctuations are observed for other traits (e.g., generation length), but none are as pronounced as the patterns for ChickPC1 and adult body mass.

Supplementary Figure 7a

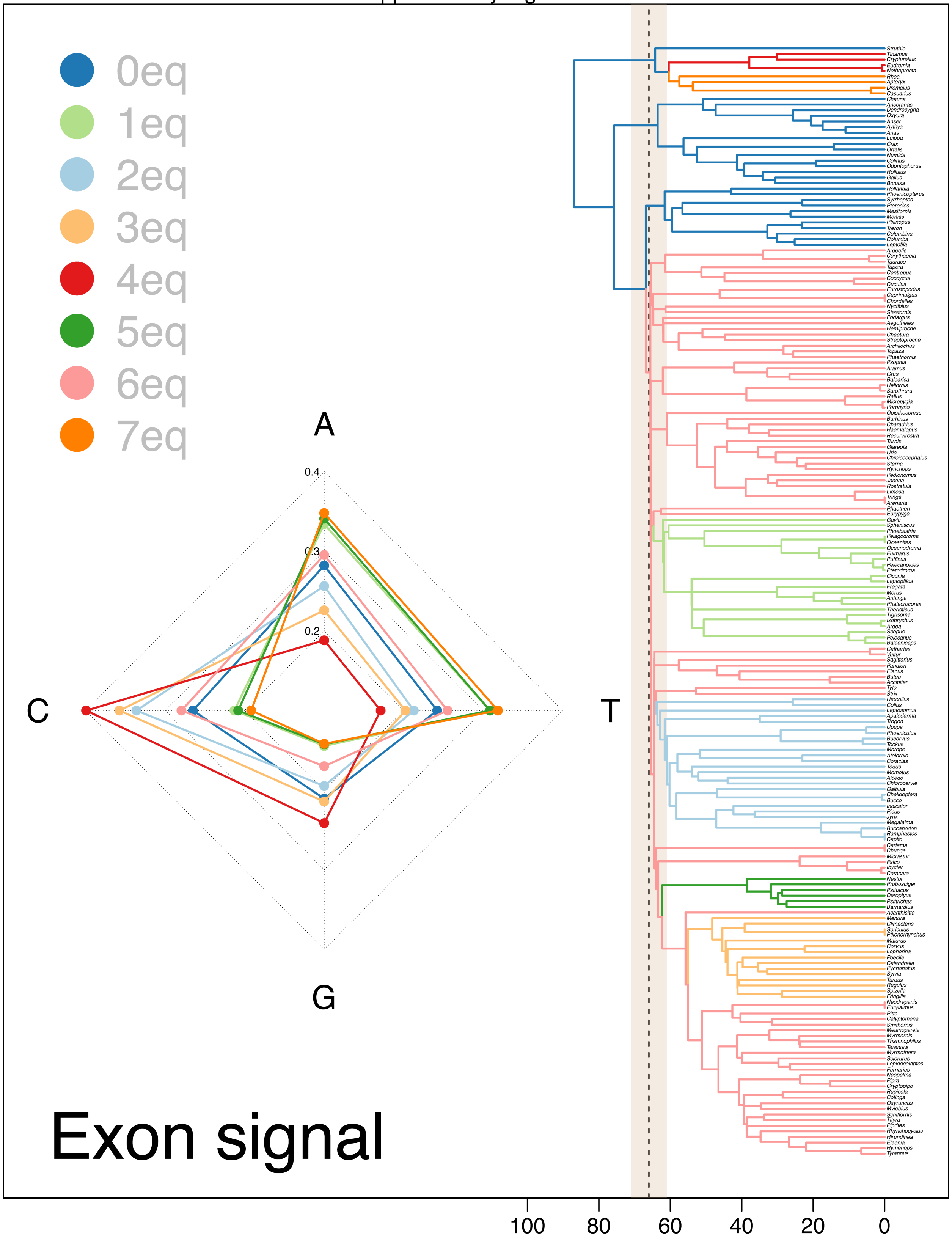

Supplementary Figure 7b

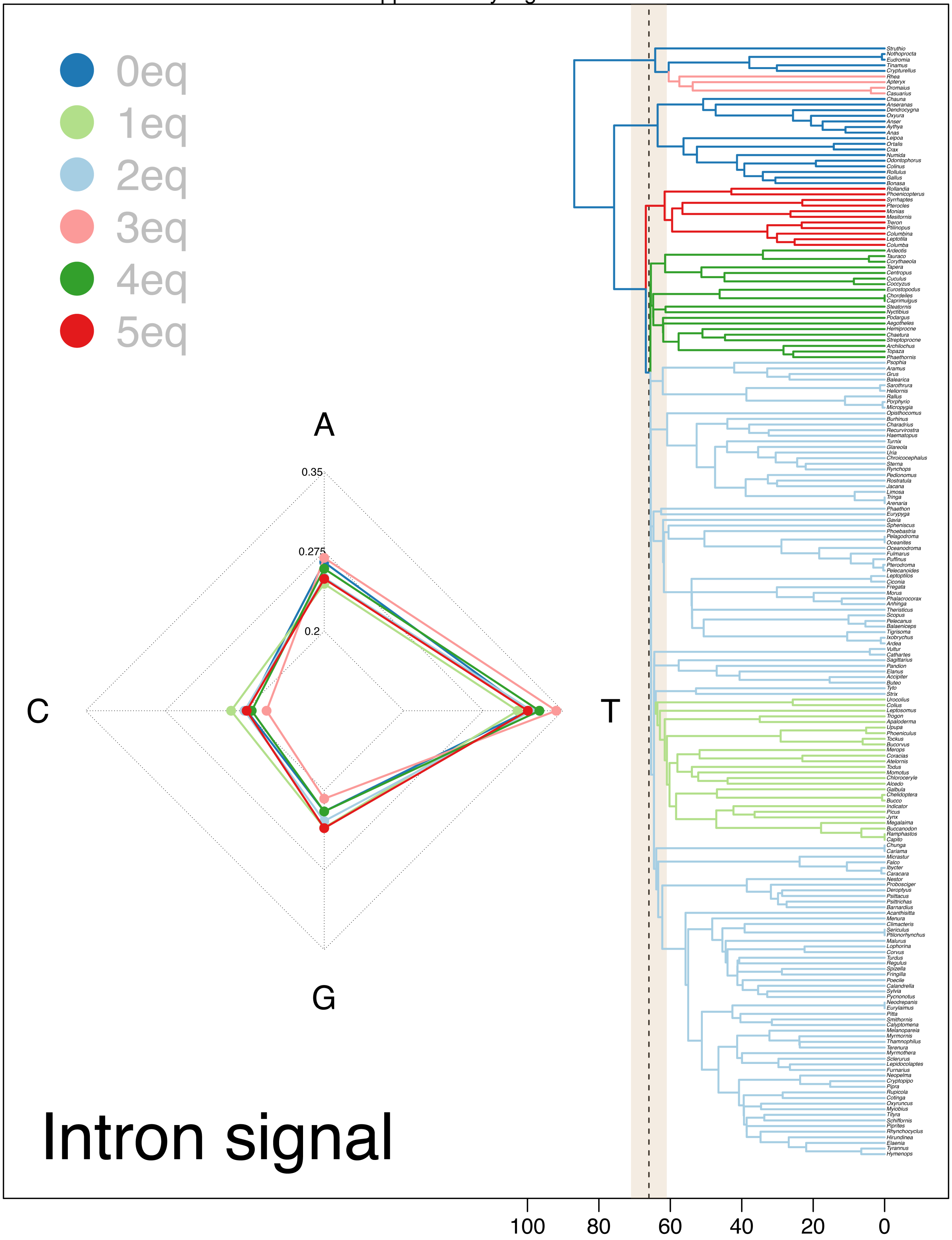

Supplementary Figure 7c

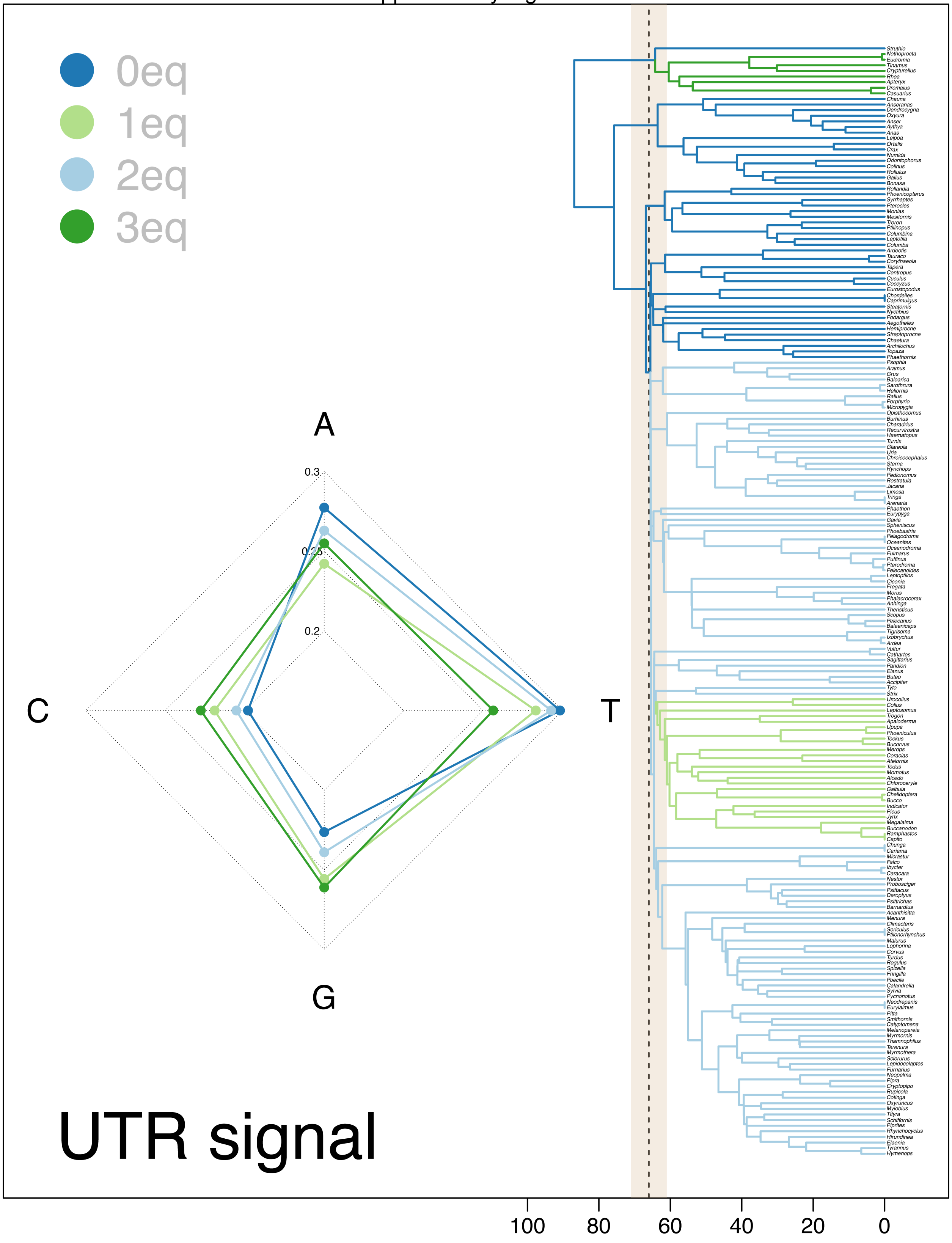

Supplementary Figure 7d

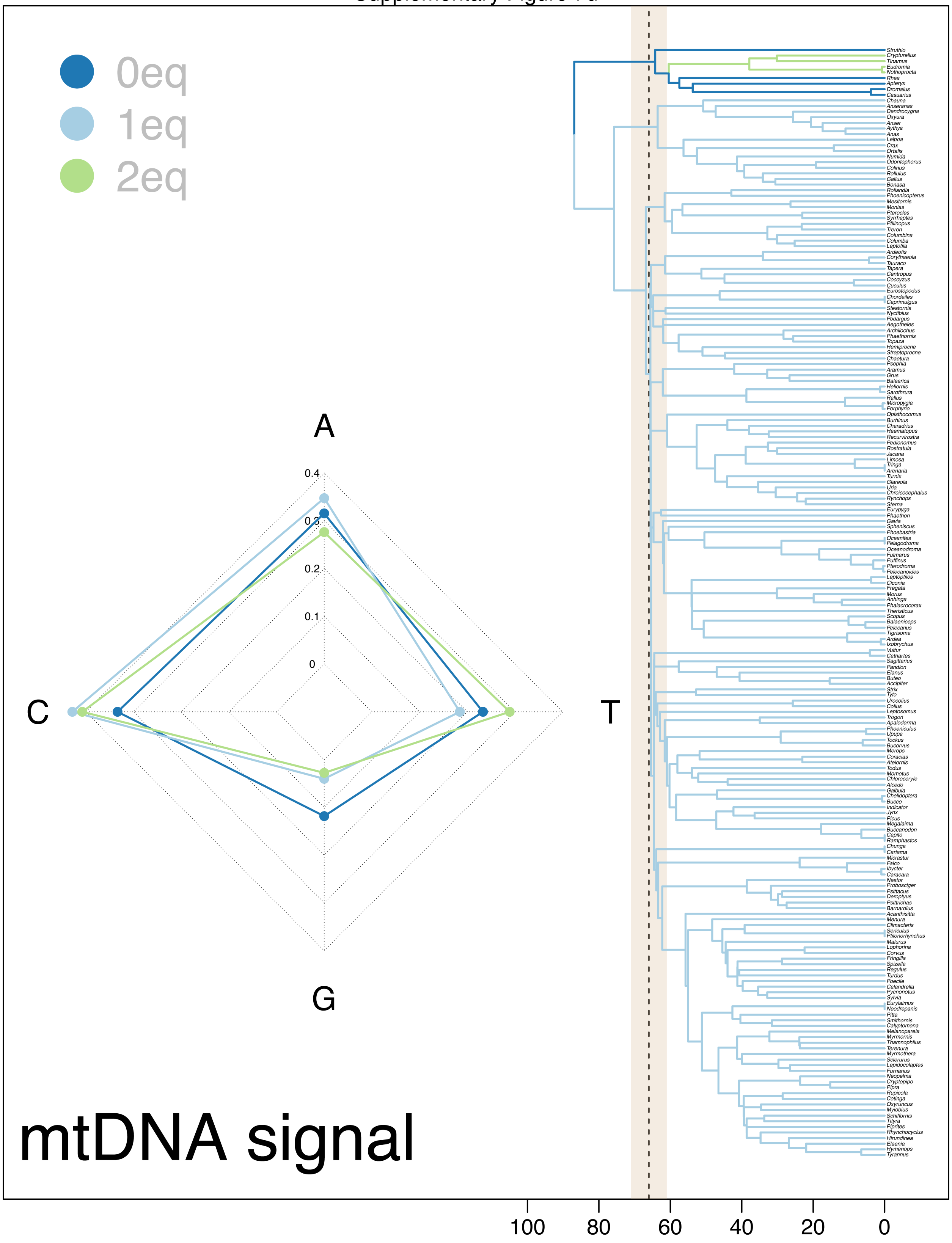

Fig. S7a-d. Maps of estimated equilibrium base frequencies for each taxon partition identified by *Janus* in analyses of different genetic data types. For each plot a-d, we show a radar plot depicting the relative estimated equilibrium base frequencies for each taxon partition. Colors match those indicated in the legend and correspond to distinct phylogenetic regimes. As noted in the main text and references to Fig. 1, the most significant deviations from empirical base frequencies are identified in our exon dataset (see main text discussion) and are concentrated around a brief interval near the end-Cretaceous mass extinction. All molecular model shifts, except for a shift in exon data in Oscine Passeriformes and mtDNA for Neognathae, are plausibly associated with lineage diversification near the K–Pg boundary (e.g., Fig. S1).

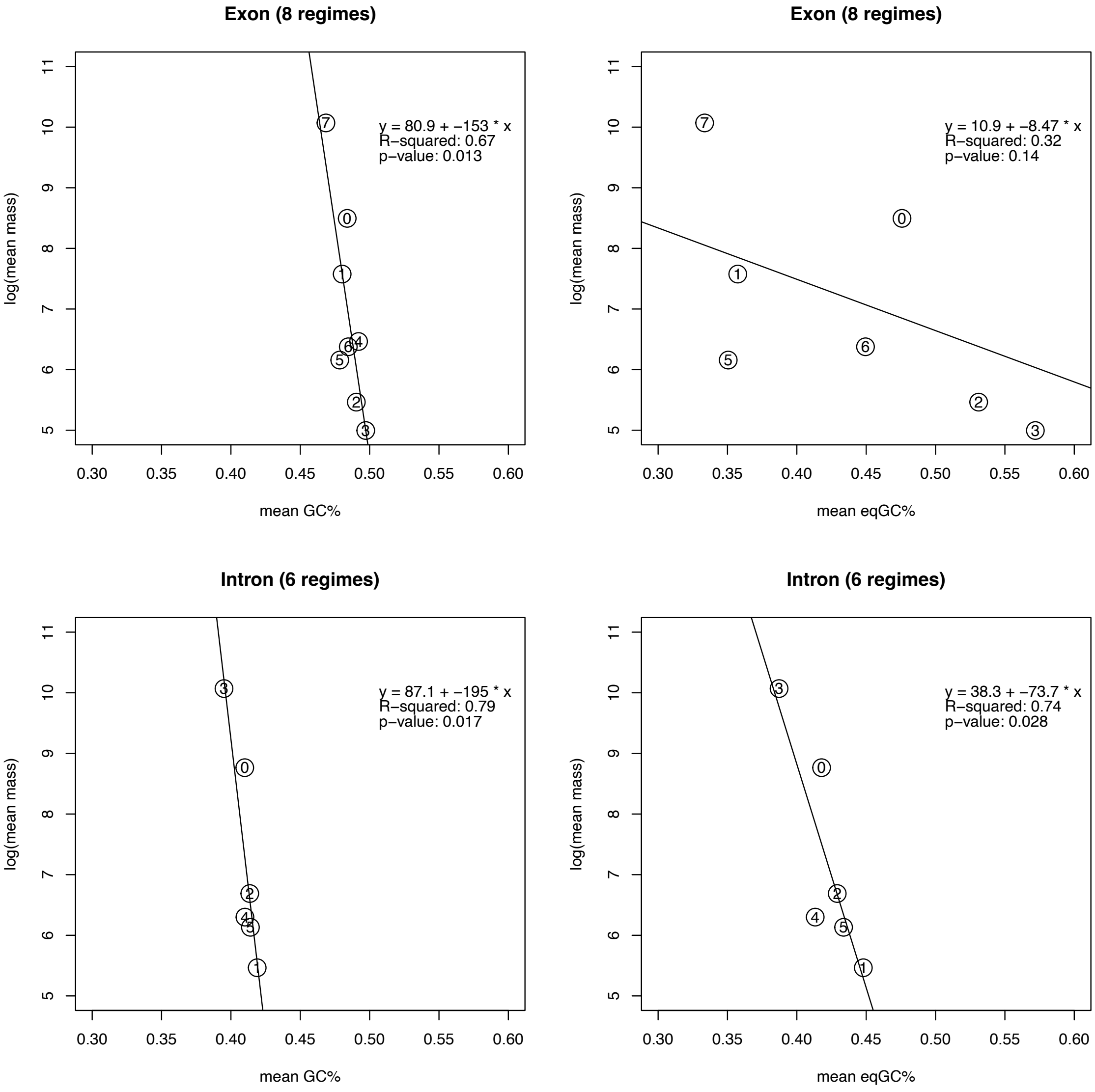

Fig. S8. GC content is strongly linked to life-history variation. In the above figure (showing linear models), we depict the relationships among average body mass within a particular macroevolutionary regime (labeled markers) identified by *Janus* against the empirical (left) or estimated equilibrium GC (right) content. We find the expected negative relationship consistent with a GC-biased gene conversion hypothesis in all cases, assuming N_e is broadly and negatively correlated with body mass (materials and methods). In ¾ of the examined cases, most of the observable variance in substitution model parameters (GC%) is explained by its association with life history ($R^2 > 0.5$). As noted in the main text, the greater deviations between empirical and estimated equilibrium base content observed for exons could be influenced by model fit related to functional constraints, codon usage bias, recombination, or selection (see supplementary analysis of codon usage, Fig. S2, S7a-d).

Statistical performance of *Janus*

Overall, we observe encouraging performance metrics consistent with those reported by Smith *et al.* (*30*). We detected only one false positive out of 1,200 datasets simulated without shifts (Fig. S9a); thus, the rate of false positives was essentially zero across deep or shallow branches.

For datasets simulated with one to four phylogenetically independent shifts (Fig. S9b-e), the average false negative rate declines to negligible levels as dataset size increases and is only significantly greater than zero in a few cases for the smallest simulated datasets of 2kbp (One-Sample t- and Z-tests, n=100, p-values below a significance level of 0.05 after correcting for the false discovery rate (*102*)). For any larger dataset (> 2kpb), the average false negative rate was never significantly larger than zero, though we note that the average false negative rate increases slowly as more shifts are added. For false positives, we observed no cases where the average false positive rate was significantly greater than zero; however, the average false positive rate (and associated standard errors) increases slightly for phylograms based on introns, UTRs, and mtDNA (not exons), as dataset size increases. We suspect this is due to weak over-fitting (*30*), which is mitigated by only considering strongly supported shifts (e.g., model weights ~ 1.0) in our analyses of empirical data. In any case, as noted above, we emphasize that there are no cases where the average false positive rate is significantly greater than zero.

For cases of nested shifts (Fig. S9f), we observe similarly encouraging performance. In all cases, the average false negative rate declines to negligible levels as dataset size increases. We observe no cases where the average false negative rate is significantly greater than zero (One-Sample t- and Z-tests, n=100, at a significance level of 0.05 after correcting for the false discovery rate). For phylograms based on exons and introns, we observe a weak increase in the average false positive rate as the dataset size increases (to about 10%). Nonetheless, we again observe no cases where the average false positive rate is significantly greater than zero. We again suspect this is due to weak overfitting, as noted above, but we are not concerned that this pattern influences our results based on empirical data, as those analyses only report strongly supported shifts.

In sum, analyses of 9,200 simulated datasets following a wide array of nonhomogeneous patterns of molecular evolution indicate that the performance of *Janus* is suitable for analyses of substitution model shifts in the context of the Avian phylogeny. In general, under-fitting (e.g., failing to detect simulated shifts) becomes negligible as dataset size increases, and overfitting is never significantly greater than zero with datasets of sufficient size. As all the empirical datasets analyzed in the present study exceed these thresholds, these potential sources of model underperformance should have minimal impact. Our simulations indicate the signal of molecular model shifts described in the main text is robust to the pattern of rapid cladogenesis observed for Aves and that molecular model shifts are only detected when empirical patterns of sequence variation are strong.

S9a

No shifts

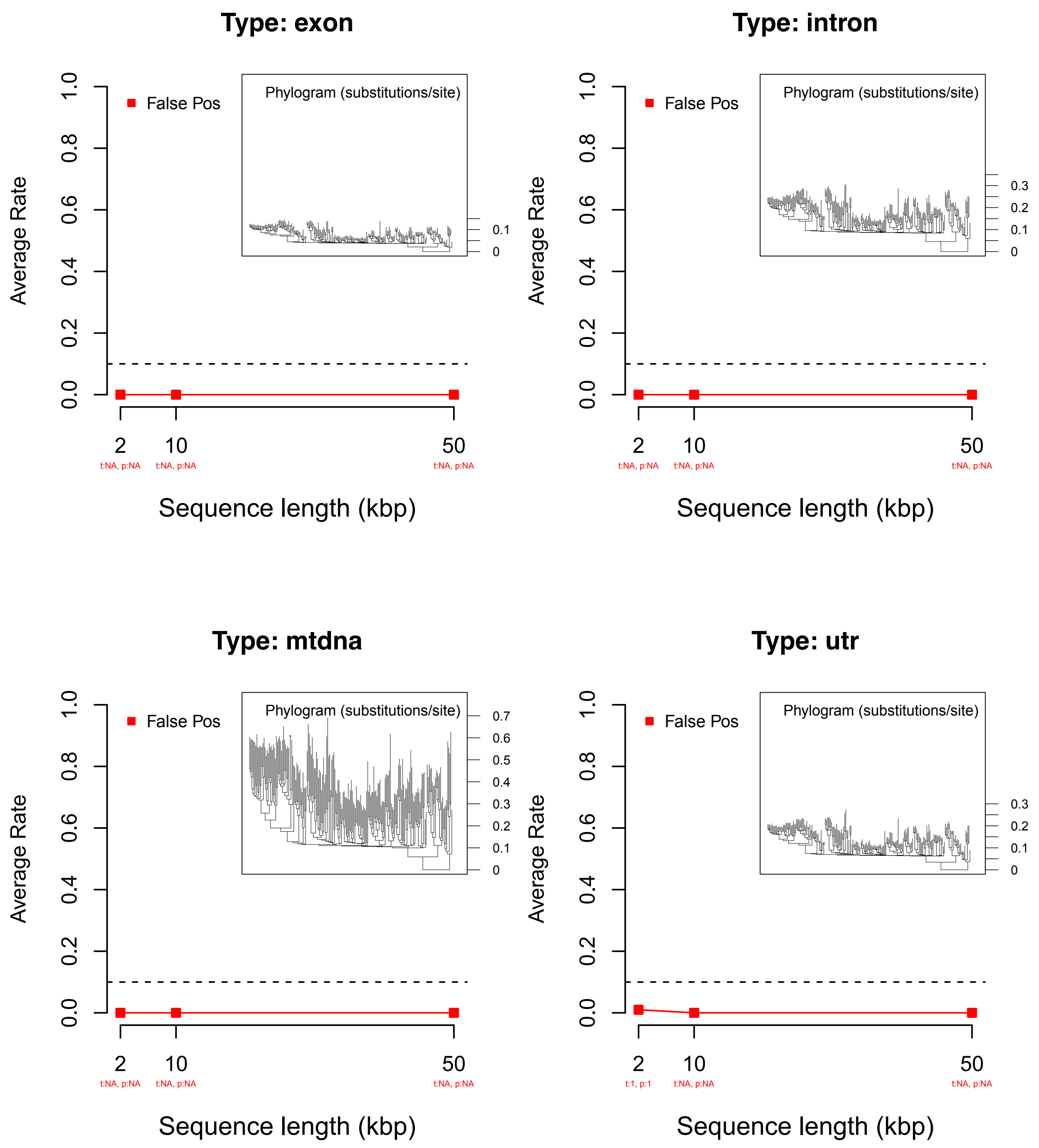

S9b

One derived shift

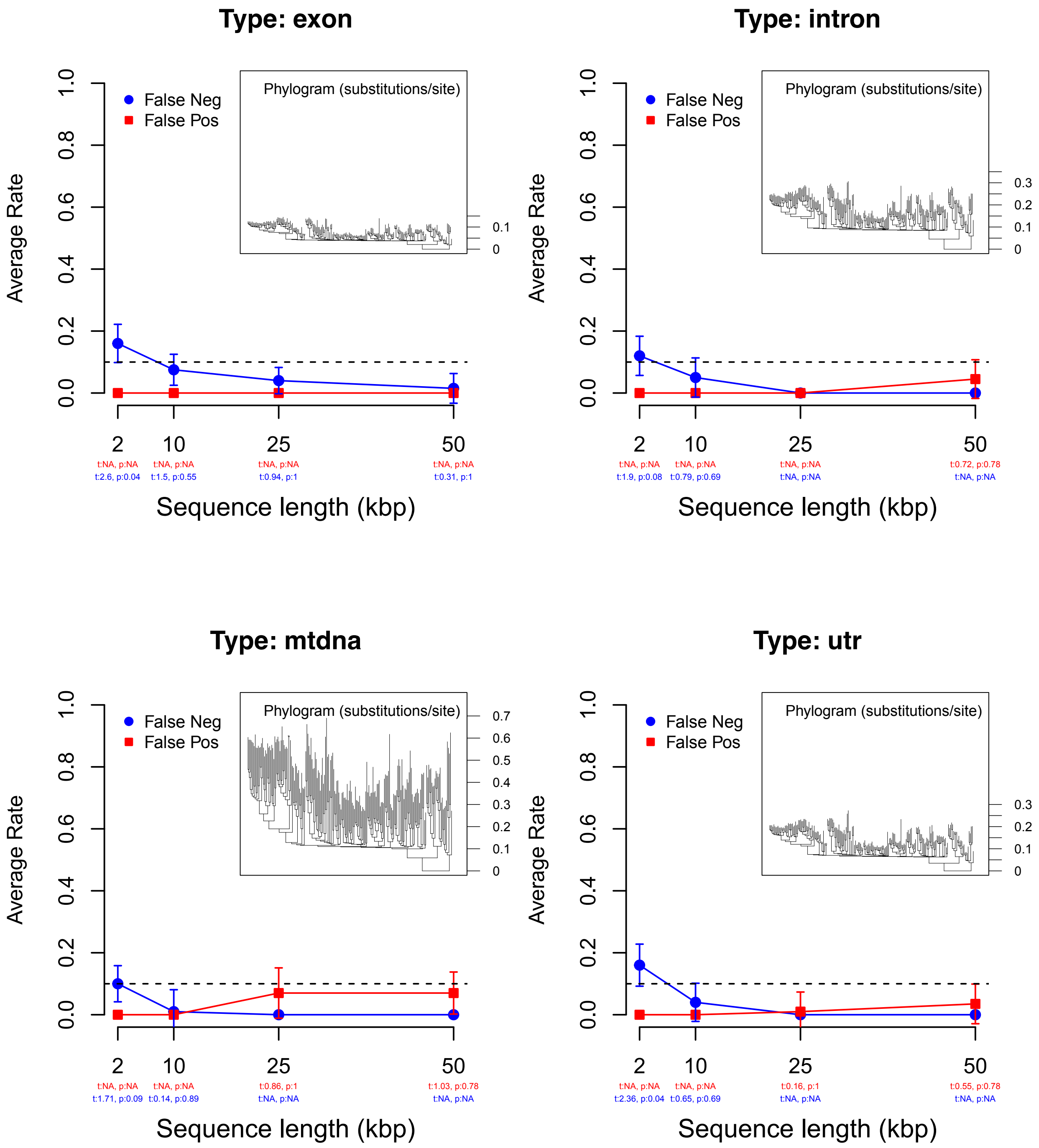

S9c

Two derived shifts (independent)

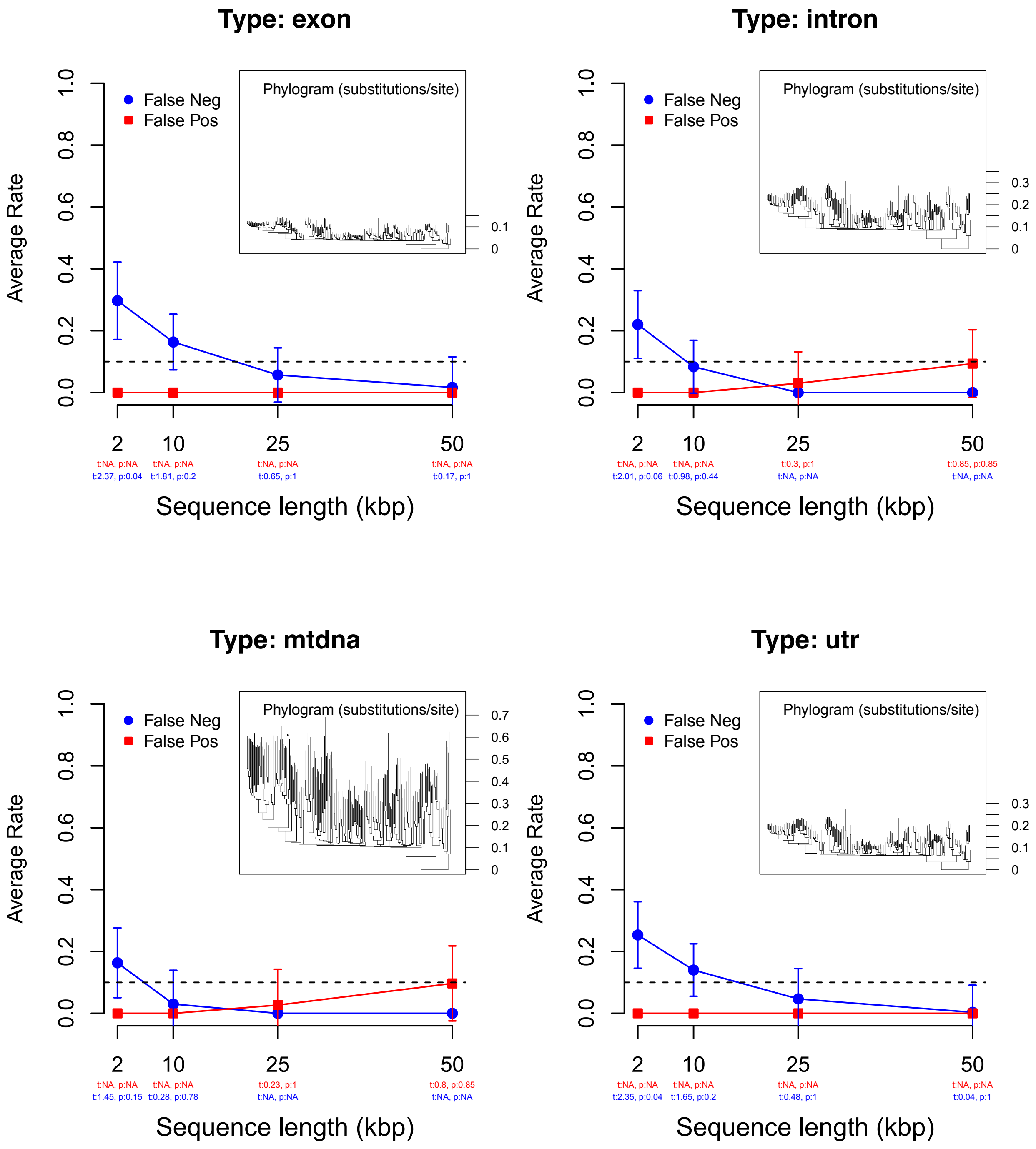

S9d

Three derived shifts (independent)

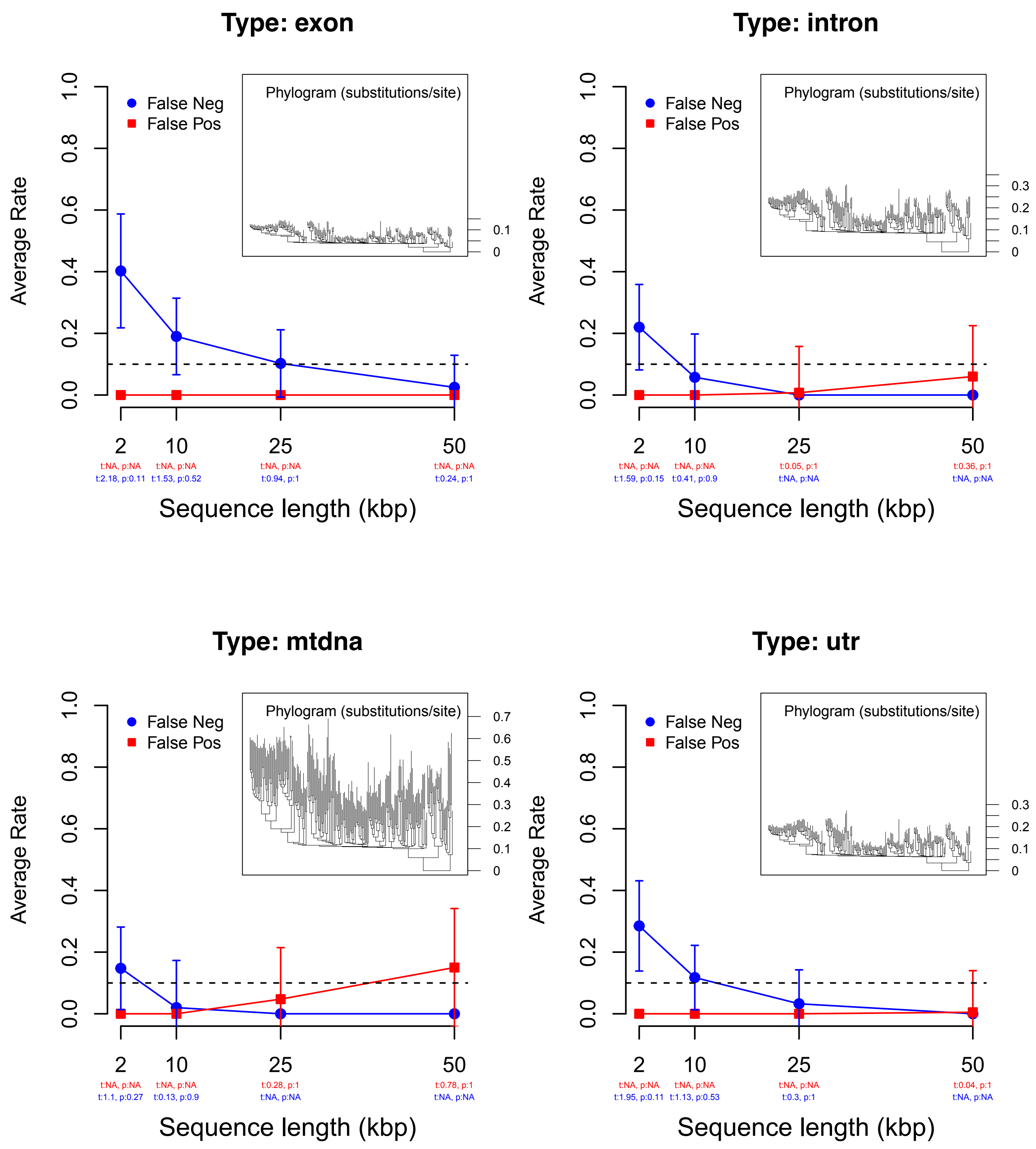

S9e

Four derived shifts (independent)

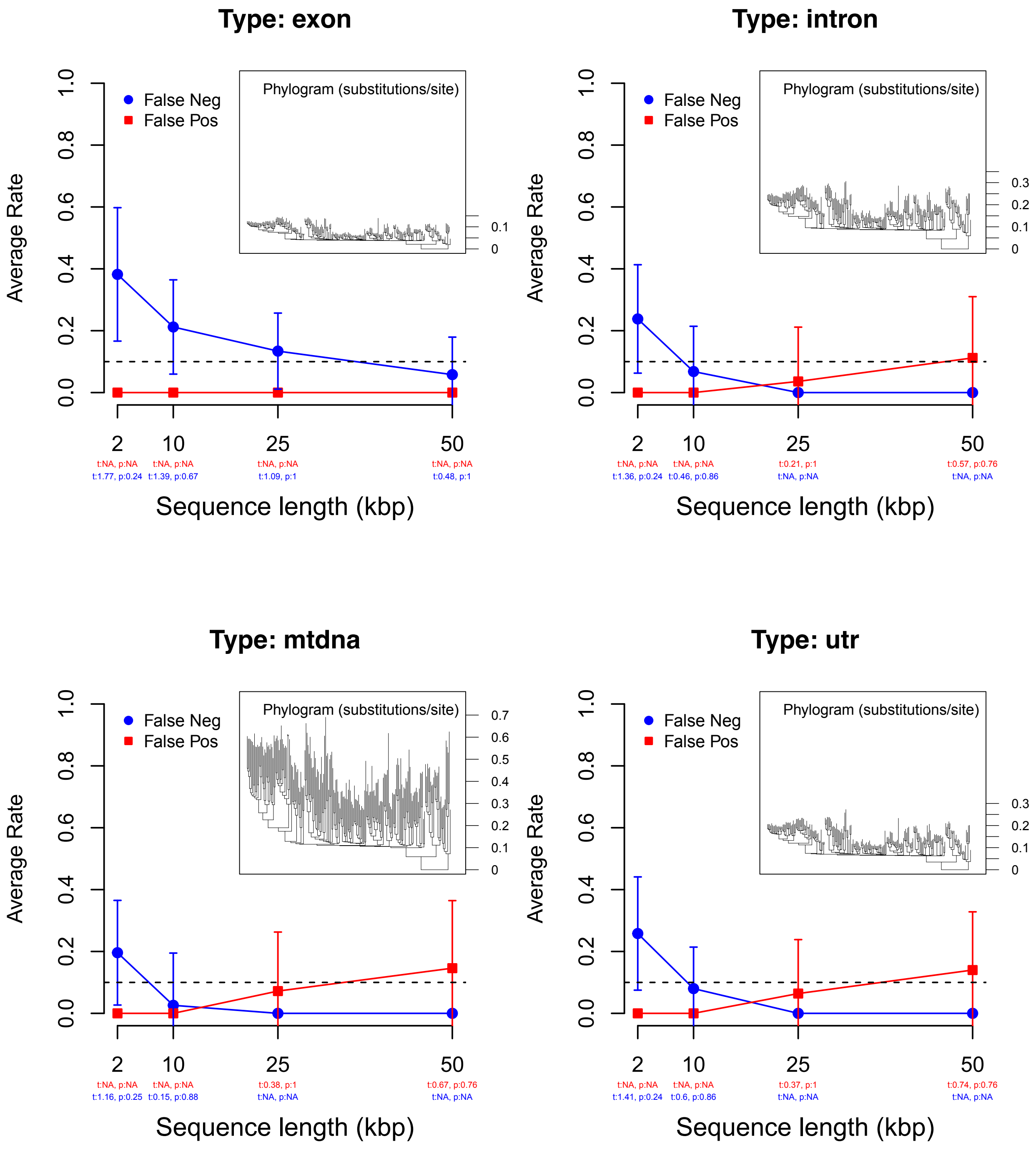

S9f

Nested shifts

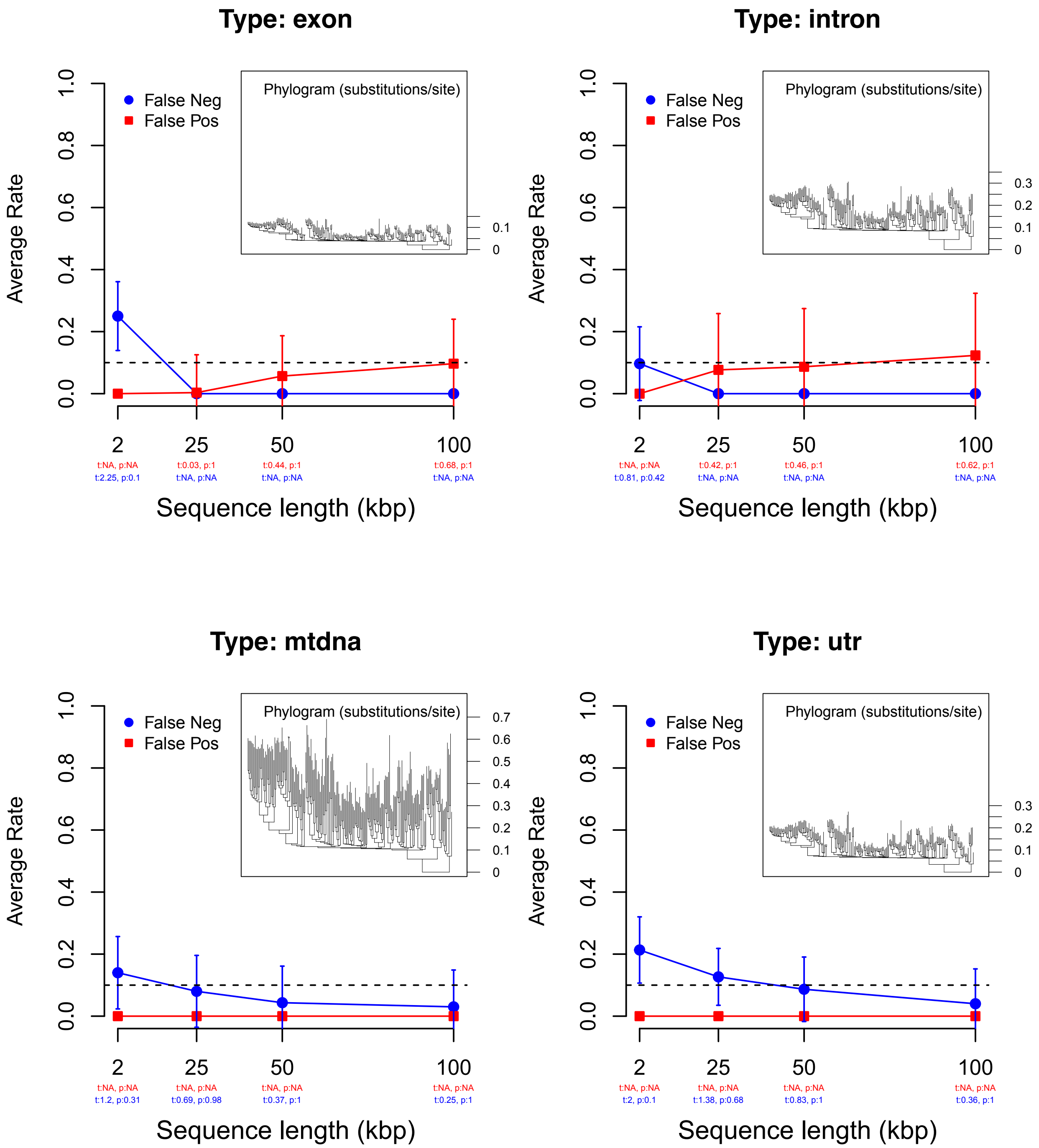

Fig. S9a-f. Summary of 9,200 simulated datasets and associated analyses used to investigate the performance of *Janus* under a wide range of non-homogeneous model shift scenarios. For each shift scenario, we plot the average false positive and negative rates for each reference phylogram, with associated standard error. Below each plot, we show the results from One-Sample t-tests (Z-tests were similar), which evaluate the null hypothesis that a given configuration and simulated dataset have an average false positive or negative rate that is not greater than zero (after controlling for the false discovery rate due to multiple tests). At a 0.05 significance threshold, few tests suggest that simulated datasets have false positive or negative rates greater than zero. A dashed line at 0.1 (10%) is included for reference. We also provide scaled phylograms to illustrate the range of relative and total branch lengths (tree shapes) evaluated.

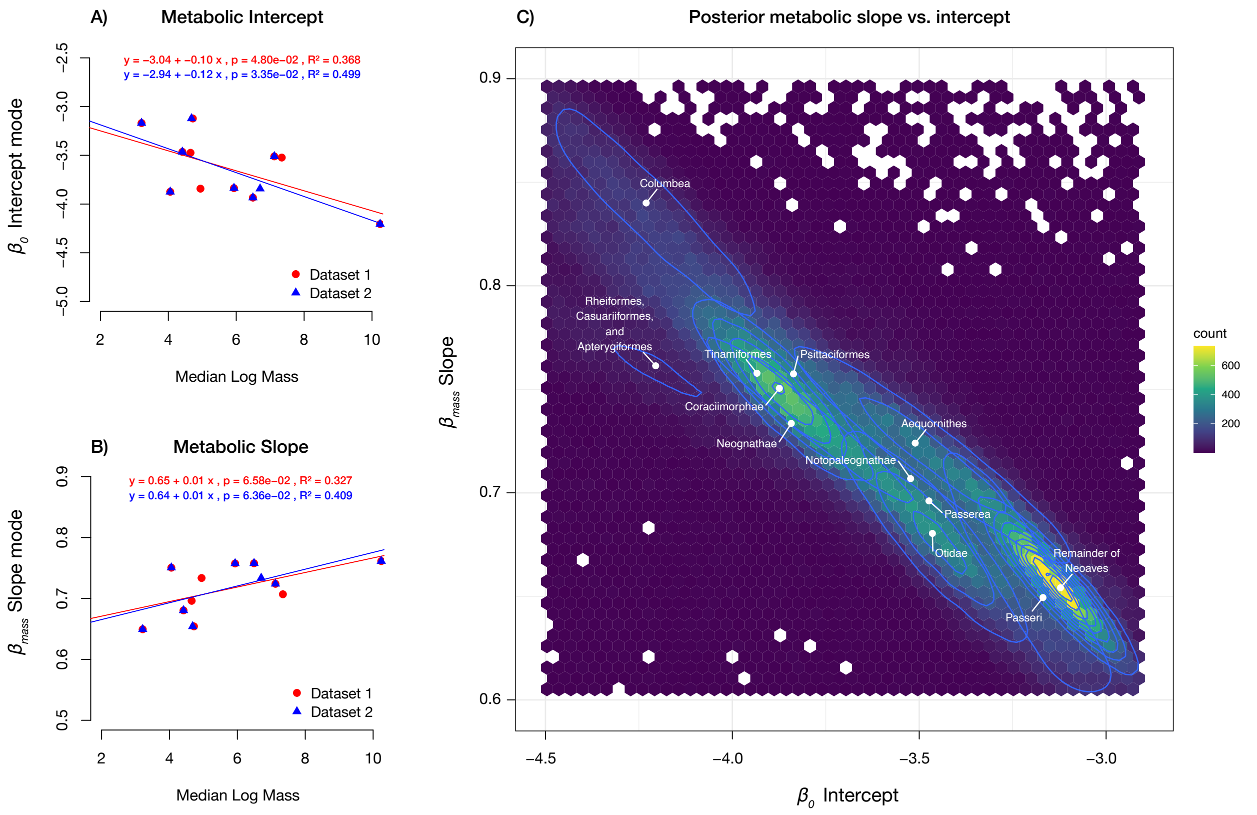

Fig. S10. Metabolic scaling parameters are generally predicted by clade mass. As shown in Fig. 3, we analyzed metabolic data under a Bayesian framework to generate posterior estimates of intercept (β_0) and slope (β_{mass}) coefficients from an evolutionary allometric regression model. Here, we depict the posterior estimates for slope and intercept across a fixed allometric shift model recapitulating molecular model regimes across all data types. On the left, we show how metabolic intercept (A, β_0) and slope (B, β_{mass}) coefficients are related to the median mass of species in each molecular model regime. Taxa with smaller mass tend to have lower slopes and higher intercept terms (see discussion) than taxa with higher mass. The labels for Datasets 1 and 2 refer to slightly different approaches for estimating group mass. Dataset 1 includes all members within each noted higher taxon, whereas Dataset 2 summarizes the masses of only the "visible" terminal members of each taxon (excluding outliers, e.g., paraphyletic groups, see Fig 1.). On the right (C), we show the posterior densities for allometric slope (β_{mass}) as a function of allometric intercept (β_0). The posterior samples in each region are depicted as a 2D-kernel density with contour lines (see supplementary R scripts in the BMR directory).

Clade	Authority	Exons	Introns	UTRs	mtDNAs	β_0	β_0 95% HPD	β_{mass}	β_{mass} 95% HPD	*pp*	Power law	ESS (β_0, β_{mass})
Aves	Linneaus, 1758	anc.	anc.	anc.	anc.	-3.81	-5.68, -1.79	0.69	0.52, 0.86	-	$0.02M^{0.69}$	4670, 4586
Paleognathae	Pycraft, 1900	-	-	-	-	-	-	-	-	-	-	
Notopaleognathae	Yuri et al. 2013	-	-	x	-	-3.52	-6.86, -0.01	0.70	0.5, 0.89	0.06	$0.03M^{0.7}$	10939, 18009
Tinamiformes	Huxley, 1872	x	-	-	x	-3.89	-4.99, -2.8	0.77	0.6, 0.94	0.10	$0.02M^{0.77}$	3002, 2980
Rheiformes, Casuariiformes, and Apterygiformes	Unnamed clade	x	x	-	-	-4.23	-5.65, -2.83	0.76	0.62, 0.91	0.15	$0.01M^{0.76}$	1573, 1556
Neognathae	Pycraft, 1900	-	-	-	x	-3.84	-4.71, -2.93	0.74	0.61, 0.87	0.02	$0.02M^{0.74}$	583, 582
Columbea	Jarvis 2014	-	x	-	-	-4.25	-4.97, -3.49	0.84	0.71, 0.98	0.39	$0.01M^{0.84}$	1694, 1693
Passerea	Jarvis 2014	x	-	-	-	-3.47	-6.82, -0.05	0.70	0.51, 0.9	0.07	$0.03M^{0.7}$	10704, 18009
Otidae	Wagler 1830	-	x	-	-	-3.46	-3.77, -3.15	0.68	0.61, 0.74	0.12	$0.03M^{0.68}$	4487, 4574
Reminader of Neoaves	Unnamed clade	-	x	x	-	-3.13	-3.32, -2.93	0.66	0.62, 0.7	0.38	$0.04M^{0.66}$	1590, 1596
Aequornithes	Jarvis 2014	x	-	-	-	-3.46	-3.98, -2.95	0.72	0.65, 0.79	0.08	$0.03M^{0.72}$	1052, 1040
Coraciimorphae	Sibley & Ahlquist, 1990	x	x	x	-	-3.87	-4.13, -3.61	0.75	0.7, 0.8	0.98	$0.02M^{0.75}$	3936, 4002
Psittaciformes	Wagler, 1830	x	-	-	-	-3.82	-4.71, -2.89	0.75	0.6, 0.91	0.11	$0.02M^{0.75}$	2572, 2593
Passeri	Linnaeus, 1758	x	-	-	-	-3.19	-3.64, -2.71	0.65	0.52, 0.79	0.04	$0.04M^{0.65}$	3531, 3530

Table S1. Summary of molecular shifts across avian higher taxa (*36, 150, 152-156*).

For each clade on which we detect a molecular model shift, we note the taxonomic authority and which data types are identified to have model shifts (with an ‘x’). We report the associated metabolic allometric parameter estimates from *bayou* for the reader’s reference: intercept (β_0), slope (β_{mass}), their associated 95% highest posterior density intervals (HPD), the posterior probability of an allometric shift occurring on a particular edge (*pp*), and the estimated power law describing the association between metabolic rate and body mass ($e^{\beta_0} \times mass^{\beta_{mass}}$). Notably, the diverse clade Coraciimoraphae is detected to have molecular model shifts in all nuclear genetic data types and maximal posterior probability for a shift in metabolic allometry. All ESS values were > 500, indicating adequate posterior sampling. Plots showing the evolution of Gelman and Rubin's shrink factor (*146*) as the number of iterations increases are provided as supplemental data (https://github.com/jakeberv/avian_molecular_shifts/tree/main/BMR).

AICc	Authority	p-value	2.50%	97.50%	Odds Ratio
Aves	Linneaus, 1758	anc.	anc.	anc.	anc.
Paleognathae	Pycraft, 1900	-	-	-	-
Notopaleognathae	Yuri et al. 2013	0.24	0.65	Inf	1.49
Tinamiformes	Huxley, 1872	0.00	8.15	Inf	13.84
Rheiformes, Casuariiformes, and Apterygiformes	Unnamed clade	0.01	1.20	Inf	2.19
Neognathae	Pycraft, 1900	0.00	14.74	Inf	25.74
Columbea	Jarvis 2014	0.00	201.35	Inf	Inf
Passerea	Jarvis 2014	0.00	16.78	Inf	29.64
Otidae	Wagler 1830	0.00	30.77	Inf	58.60
Reminader of Neoaves	Unnamed clade	0.00	1.96	Inf	3.58
Aequornithes	Jarvis 2014	0.00	201.35	Inf	Inf
Coraciimorphae	Sibley & Ahlquist, 1990	0.00	164.74	Inf	Inf
Psittaciformes	Wagler, 1830	0.00	201.35	Inf	Inf
Passeri	Linnaeus, 1758	0.00	169.99	Inf	Inf

pBIC	Authority	p-value	2.50%	97.50%	Odds Ratio
Aves	Linneaus, 1758	anc.	anc.	anc.	anc.
Paleognathae	Pycraft, 1900	-	-	-	-
Notopaleognathae	Yuri et al. 2013	0.04	1.04	Inf	2.07
Tinamiformes	Huxley, 1872	0.00	2.03	Inf	3.31
Rheiformes, Casuariiformes, and Apterygiformes	Unnamed clade	0.00	2.76	Inf	4.72
Neognathae	Pycraft, 1900	0.00	3.41	Inf	5.43
Columbea	Jarvis 2014	0.00	469.25	Inf	Inf
Passerea	Jarvis 2014	0.00	504.78	Inf	2945.38
Otidae	Wagler 1830	0.78	0.03	Inf	0.72
Reminader of Neoaves	Unnamed clade	0.78	0.10	Inf	0.72
Aequornithes	Jarvis 2014	0.00	679.55	Inf	Inf
Coraciimorphae	Sibley & Ahlquist, 1990	0.00	172.19	Inf	634.71
Psittaciformes	Wagler, 1830	0.00	53.72	Inf	122.13
Passeri	Linnaeus, 1758	1.00	0.00	Inf	0.00

Table S2. Summary of Fisher's exact test for shifts in life-history trait optima associated with molecular model shifts and higher taxa (*36, 150, 152-156*).

With ℓ1ou, we used parametric bootstrapping to estimate the frequency of shift detections and the null false positive rate for each candidate edge under simulated multivariate Brownian motion. We use Fisher's test to ask if the proportion of empirical shift detections in the bootstrapped dataset is higher than that of null false positives in the simulated dataset. All candidate edges are supported under AICc or pBIC criteria, but not both in a few cases.

Table S3. Details of mtDNA dataset assembly.

Provided as a separate file. Also, see supplemental data at https://github.com/jakeberv/avian_molecular_shifts

Table S4. Life-history and metabolic rate datasets.

Provided as a separate file. Also, see supplemental data at https://github.com/jakeberv/avian_molecular_shifts/tree/main/LHT_DATA

REFERENCES AND NOTES

1. L. W. Alvarez, W. Alvarez, F. Asaro, H. V. Michel, Extraterrestrial Cause for the Cretaceous-Tertiary Extinction. *Science* **208**, 1095-1108 (1980).

2. A. A. Chiarenza, A. Farnsworth, P. D. Mannion, D. J. Lunt, P. J. Valdes, J. V. Morgan, P. A. Allison, Asteroid impact, not volcanism, caused the end-Cretaceous dinosaur extinction. *Proceedings of the National Academy of Sciences* **117**, 17084-17093 (2020).

3. S. L. Brusatte, R. J. Butler, P. M. Barrett, M. T. Carrano, D. C. Evans, G. T. Lloyd, P. D. Mannion, M. A. Norell, D. J. Peppe, P. Upchurch, T. E. Williamson, The extinction of the dinosaurs. *Biological Reviews* **90**, 628-642 (2015).

4. P. J. Harries, P. O. Knorr, What does the 'Lilliput Effect' mean? *Palaeogeography, Palaeoclimatology, Palaeoecology* **284**, 4-10 (2009).

5. M. Friedman, Ecomorphological selectivity among marine teleost fishes during the end-Cretaceous extinction. *Proceedings of the National Academy of Sciences* **106**, 5218-5223 (2009).

6. N. R. Longrich, T. Tokaryk, D. J. Field, Mass extinction of birds at the Cretaceous–Paleogene (K–Pg) boundary. *Proceedings of the National Academy of Sciences* **108**, 15253-15257 (2011).

7. N. R. Longrich, B.-A. S. Bhullar, J. A. Gauthier, A transitional snake from the late Cretaceous period of North America. *Nature* **488**, (2012).

8. L. A. Wiest, W. E. Lukens, D. J. Peppe, S. G. Driese, J. Tubbs, Terrestrial evidence for the Lilliput effect across the Cretaceous-Paleogene (K-Pg) boundary. *Palaeogeography, Palaeoclimatology, Palaeoecology* **491**, 161-169 (2018).

9. L. Bromham, "Causes of Variation in the Rate of Molecular Evolution" in *The Molecular Evolutionary Clock* (Springer, 2020), pp. 45-64.

10. D. J. Field, J. S. Berv, A. Y. Hsiang, R. Lanfear, M. J. Landis, A. Dornburg, "Chapter 5: Timing the Extant Avian Radiation: The Rise of Modern Birds, and the Importance of Modeling Molecular Rate Variation" in *Pennaraptoran Theropod Dinosaurs Past Progress and New Frontiers,* M. Pittman, X. Xu, Eds. (Bulletin of the American Museum of Natural History, New York, 2020), vol. 440, pp. 159-181.

11. J. S. Berv, D. J. Field, Genomic Signature of an Avian Lilliput Effect across the K-Pg Extinction. *Systematic Biology* **67**, 1-13 (2018).

12. J. Stiller, S. Feng, A.-A. Chowdhury, I. Rivas-González, D. A. Duchêne, Q. Fang, Y. Deng, A. Kozlov, A. Stamatakis, S. Claramunt, J. M. T. Nguyen, S. Y. W. Ho, B. C. Faircloth, J. Haag, P. Houde, J. Cracraft, M. Balaban, U. Mai, G. Chen, R. Gao, C. Zhou, Y. Xie, Z. Huang, Z. Cao, Z. Yan, H. A. Ogilvie, L. Nakhleh, B. Lindow, B. Morel, J. Fjeldså, P. A. Hosner, R. R. da Fonseca, B. Petersen, J. A. Tobias, T. Székely, J. D. Kennedy, A. H. Reeve, A. Liker, M. Stervander, A. Antunes, D. T. Tietze, M. F. Bertelsen, F. Lei, C. Rahbek, G. R. Graves, M. H. Schierup, T.

Warnow, E. L. Braun, M. T. P. Gilbert, E. D. Jarvis, S. Mirarab, G. Zhang, Complexity of avian evolution revealed by family-level genomes. *Nature*, (2024).

13. K. Vanneste, G. Baele, S. Maere, Y. Van de Peer, Analysis of 41 plant genomes supports a wave of successful genome duplications in association with the Cretaceous–Paleogene boundary. *Genome Research* **24**, 1334-1347 (2014).

14. P. Houde, E. L. Braun, L. Zhou, Deep-Time Demographic Inference Suggests Ecological Release as Driver of Neoavian Adaptive Radiation. *Diversity* **12**, 164 (2020).

15. M. Rho, M. Zhou, X. Gao, S. Kim, H. Tang, M. Lynch, Independent Mammalian Genome Contractions Following the KT Boundary. *Genome Biology and Evolution* **1**, 2-12 (2009).

16. J. Romiguier, V. Ranwez, E. J. P. Douzery, N. Galtier, Genomic evidence for large, long-lived ancestors to placental mammals. *Mol Biol Evol* **30**, (2013).

17. C. C. Morgan, P. G. Foster, A. E. Webb, D. Pisani, J. O. McInerney, M. J. O'Connell, Heterogeneous Models Place the Root of the Placental Mammal Phylogeny. *Molecular Biology and Evolution* **30**, 2145-2156 (2013).

18. R. S. Waples, G. Luikart, J. R. Faulkner, D. A. Tallmon, Simple life-history traits explain key effective population size ratios across diverse taxa. *Proceedings of the Royal Society B: Biological Sciences* **280**, 20131339 (2013).

19. Van M. Savage, James F. Gillooly, James H. Brown, Geoffrey B. West, Eric L. Charnov, Effects of Body Size and Temperature on Population Growth. *The American Naturalist* **163**, 429-441 (2004).

20. S. V. Edwards, B. Fertil, A. Giron, P. J. Deschavanne, A Genomic Schism in Birds Revealed by Phylogenetic Analysis of DNA Strings. *Systematic Biology* **51**, 599-613 (2002).

21. C. C. Weber, B. Boussau, J. Romiguier, E. D. Jarvis, H. Ellegren, Evidence for GC-biased gene conversion as a driver of between-lineage differences in avian base composition. *Genome Biology* **15**, 549 (2014).

22. B. Nabholz, A. Künstner, R. Wang, E. D. Jarvis, H. Ellegren, Dynamic evolution of base composition: causes and consequences in avian phylogenomics. *Mol Biol Evol* **28**, (2011).

23. A. Dornburg, M. C. Brandley, M. R. McGowen, T. J. Near, Relaxed Clocks and Inferences of Heterogeneous Patterns of Nucleotide Substitution and Divergence Time Estimates across Whales and Dolphins (Mammalia: Cetacea). *Molecular Biology and Evolution* **29**, 721-736 (2012).

24. P. G. Foster, Modeling Compositional Heterogeneity. *Systematic Biology* **53**, 485-495 (2004).

25. A. M. Ritchie, T. L. Stark, D. A. Liberles, Inferring the number and position of changes in selective regime in a non-equilibrium mutation-selection framework. *BMC Ecology and Evolution* **21**, 39 (2021).

26. J. Romiguier, E. Figuet, N. Galtier, E. J. P. Douzery, B. Boussau, J. Y. Dutheil, V. Ranwez, Fast and robust characterization of time-heterogeneous sequence evolutionary processes using substitution mapping. *PLoS One* **7**, (2012).

27. J. M. Beaulieu, D.-C. Jhwueng, C. Boettiger, B. C. O'Meara, Modeling stabilizing selection: expanding the Ornstein-Uhlenbeck model of adaptive evolution. *Evolution* **66**, 2369-2383 (2012).

28. M. Khabbazian, R. Kriebel, K. Rohe, C. Ané, Fast and accurate detection of evolutionary shifts in Ornstein–Uhlenbeck models. *Methods in Ecology and Evolution* **7**, 811-824 (2016).

29. J. C. Uyeda, M. W. Pennell, E. T. Miller, R. Maia, C. R. McClain, The Evolution of Energetic Scaling across the Vertebrate Tree of Life. *The American Naturalist* **190**, 185-199 (2017).

30. S. A. Smith, N. Walker-Hale, C. T. Parins-Fukuchi, Compositional shifts associated with major evolutionary transitions in plants. *New Phytologist* **239**, 2404-2415 (2023).

31. G. A. Bravo, C. J. Schmitt, S. V. Edwards, What Have We Learned from the First 500 Avian Genomes? *Annual Review of Ecology, Evolution, and Systematics* **52**, 611-639 (2021).

32. S. Feng, J. Stiller, Y. Deng, J. Armstrong, Q. Fang, A. H. Reeve, D. Xie, G. Chen, C. Guo, B. C. Faircloth, B. Petersen, Z. Wang, Q. Zhou, M. Diekhans, W. Chen, S. Andreu-Sánchez, A. Margaryan, J. T. Howard, C. Parent, G. Pacheco, M.-H. S. Sinding, L. Puetz, E. Cavill, Â. M. Ribeiro, L. Eckhart, J. Fjeldså, P. A. Hosner, R. T. Brumfield, L. Christidis, M. F. Bertelsen, T. Sicheritz-Ponten, D. T. Tietze, B. C. Robertson, G. Song, G. Borgia, S. Claramunt, I. J. Lovette, S. J. Cowen, P. Njoroge, J. P. Dumbacher, O. A. Ryder, J. Fuchs, M. Bunce, D. W. Burt, J. Cracraft, G. Meng, S. J. Hackett, P. G. Ryan, K. A. Jønsson, I. G. Jamieson, R. R. da Fonseca, E. L. Braun, P. Houde, S. Mirarab, A. Suh, B. Hansson, S. Ponnikas, H. Sigeman, M. Stervander, P. B. Frandsen, H. van der Zwan, R. van der Sluis, C. Visser, C. N. Balakrishnan, A. G. Clark, J. W. Fitzpatrick, R. Bowman, N. Chen, A. Cloutier, T. B. Sackton, S. V. Edwards, D. J. Foote, S. B. Shakya, F. H. Sheldon, A. Vignal, A. E. R. Soares, B. Shapiro, J. González-Solís, J. Ferrer-Obiol, J. Rozas, M. Riutort, A. Tigano, V. Friesen, L. Dalén, A. O. Urrutia, T. Székely, Y. Liu, M. G. Campana, A. Corvelo, R. C. Fleischer, K. M. Rutherford, N. J. Gemmell, N. Dussex, H. Mouritsen, N. Thiele, K. Delmore, M. Liedvogel, A. Franke, M. P. Hoeppner, O. Krone, A. M. Fudickar, B. Milá, E. D. Ketterson, A. E. Fidler, G. Friis, Á. M. Parody-Merino, P. F. Battley, M. P. Cox, N. C. B. Lima, F. Prosdocimi, T. L. Parchman, B. A. Schlinger, B. A. Loiselle, J. G. Blake, H. C. Lim, L. B. Day, M. J. Fuxjager, M. W. Baldwin, M. J. Braun, M. Wirthlin, R. B. Dikow, T. B. Ryder, G. Camenisch, L. F. Keller, J. M. DaCosta, M. E. Hauber, M. I. M. Louder, C. C. Witt, J. A. McGuire, J. Mudge, L. C. Megna, M. D. Carling, B. Wang, S. A. Taylor, G. Del-Rio, A. Aleixo, A. T. R. Vasconcelos, C. V. Mello, J. T. Weir, D. Haussler, Q. Li, H. Yang, J. Wang, F. Lei, C. Rahbek, M. T. P. Gilbert, G. R. Graves, E. D. Jarvis, B. Paten, G. Zhang, Dense sampling of bird diversity increases power of comparative genomics. *Nature* **587**, 252-257 (2020).

33. R. Maor, T. Dayan, H. Ferguson-Gow, K. E. Jones, Temporal niche expansion in mammals from a nocturnal ancestor after dinosaur extinction. *Nature Ecology & Evolution* **1**, 1889-1895 (2017).

34. J. J. Hughes, J. S. Berv, S. G. B. Chester, E. J. Sargis, D. J. Field, Ecological selectivity and the evolution of mammalian substrate preference across the K–Pg boundary. *Ecology and Evolution* **11**, 14540-14554 (2021).

35. D. J. Field, A. Bercovici, J. S. Berv, R. Dunn, D. E. Fastovsky, T. R. Lyson, V. Vajda, J. A. Gauthier, Early Evolution of Modern Birds Structured by Global Forest Collapse at the End-Cretaceous Mass Extinction. *Current Biology* **28**, 1825-1831.e1822 (2018).

36. E. D. Jarvis, S. Mirarab, A. J. Aberer, B. Li, P. Houde, C. Li, S. Y. W. Ho, B. C. Faircloth, B. Nabholz, J. T. Howard, A. Suh, C. C. Weber, R. R. da Fonseca, J. Li, F. Zhang, H. Li, L. Zhou, N. Narula, L. Liu, G. Ganapathy, B. Boussau, M. S. Bayzid, V. Zavidovych, S. Subramanian, T. Gabaldón, S. Capella-Gutiérrez, J. Huerta-Cepas, B. Rekepalli, K. Munch, M. Schierup, B. Lindow, W. C. Warren, D. Ray, R. E. Green, M. W. Bruford, X. Zhan, A. Dixon, S. Li, N. Li, Y. Huang, E. P. Derryberry, M. F. Bertelsen, F. H. Sheldon, R. T. Brumfield, C. V. Mello, P. V. Lovell, M. Wirthlin, M. P. C. Schneider, F. Prosdocimi, J. A. Samaniego, A. M. V. Velazquez, A. Alfaro-Núñez, P. F. Campos, B. Petersen, T. Sicheritz-Ponten, A. Pas, T. Bailey, P. Scofield, M. Bunce, D. M. Lambert, Q. Zhou, P. Perelman, A. C. Driskell, B. Shapiro, Z. Xiong, Y. Zeng, S. Liu, Z. Li, B. Liu, K. Wu, J. Xiao, X. Yinqi, Q. Zheng, Y. Zhang, H. Yang, J. Wang, L. Smeds, F. E. Rheindt, M. Braun, J. Fjeldsa, L. Orlando, F. K. Barker, K. A. Jønsson, W. Johnson, K.-P. Koepfli, S. O'Brien, D. Haussler, O. A. Ryder, C. Rahbek, E. Willerslev, G. R. Graves, T. C. Glenn, J. McCormack, D. Burt, H. Ellegren, P. Alström, S. V. Edwards, A. Stamatakis, D. P. Mindell, J. Cracraft, E. L. Braun, T. Warnow, W. Jun, M. T. P. Gilbert, G. Zhang, Whole-genome analyses resolve early branches in the tree of life of modern birds. *Science* **346**, 1320-1331 (2014).

37. R. O. Prum, J. S. Berv, A. Dornburg, D. J. Field, J. P. Townsend, E. M. Lemmon, A. R. Lemmon, A comprehensive phylogeny of birds (Aves) using targeted next-generation DNA sequencing. *Nature* **526**, 569-573 (2015).

38. S. Claramunt, J. Cracraft, A new time tree reveals Earth history's imprint on the evolution of modern birds. *Science Advances* **1**, (2015).

39. R. T. Kimball, C. H. Oliveros, N. Wang, N. D. White, F. K. Barker, D. J. Field, D. T. Ksepka, R. T. Chesser, R. G. Moyle, M. J. Braun, R. T. Brumfield, B. C. Faircloth, B. T. Smith, E. L. Braun, A Phylogenomic Supertree of Birds. *Diversity* **11**, 109 (2019).

40. S. Ducatez, D. J. Field, Disentangling the avian altricial-precocial spectrum: Quantitative assessment of developmental mode, phylogenetic signal, and dimensionality. *Evolution* **75**, 2717-2735 (2021).

41. C. R. White, L. A. Alton, C. L. Bywater, E. J. Lombardi, D. J. Marshall, Metabolic scaling is the product of life-history optimization. *Science* **377**, 834-839 (2022).

42. P. S. Dodds, D. H. Rothman, J. S. Weitz, Re-examination of the "3/4-law" of Metabolism. *Journal of Theoretical Biology* **209**, 9-27 (2001).

43. Annette E. Sieg, Michael P. O'Connor, James N. McNair, Bruce W. Grant, Salvatore J. Agosta, Arthur E. Dunham, Mammalian Metabolic Allometry: Do Intraspecific Variation, Phylogeny, and Regression Models Matter? *The American Naturalist* **174**, 720-733 (2009).

44. A. A. Heusner, Size and power in mammals. *Journal of Experimental Biology* **160**, 25-54 (1991).

45. T. Kolokotrones, S. Van, E. J. Deeds, W. Fontana, Curvature in metabolic scaling. *Nature* **464**, 753-756 (2010).

46. M. Schweizer, S. T. Hertwig, O. Seehausen, Diversity versus disparity and the role of ecological opportunity in a continental bird radiation. *Journal of Biogeography* **41**, 1301-1312 (2014).

47. L. J. Harmon, J. A. Schulte, A. Larson, J. B. Losos, Tempo and Mode of Evolutionary Radiation in Iguanian Lizards. *Science* **301**, 961-964 (2003).

48. R. E. Ricklefs, R. E. Shea, I.-H. Choi, INVERSE RELATIONSHIP BETWEEN FUNCTIONAL MATURITY AND EXPONENTIAL GROWTH RATE OF AVIAN SKELETAL MUSCLE: A CONSTRAINT ON EVOLUTIONARY RESPONSE. *Evolution* **48**, 1080-1088 (1994).

49. D. Schluter, *The ecology of adaptive radiation* (OUP Oxford, 2000).

50. G. G. Simpson, *Tempo and mode in evolution* (Columbia University Press, 1944).

51. S. J. Gould, *The structure of evolutionary theory* (Harvard University Press, 2002).

52. T. J. Givnish, Adaptive radiation versus 'radiation' and 'explosive diversification': why conceptual distinctions are fundamental to understanding evolution. *New Phytologist* **207**, 297-303 (2015).

53. J. P. Drury, J. Clavel, J. A. Tobias, J. Rolland, C. Sheard, H. Morlon, Limited ecological opportunity influences the tempo of morphological evolution in birds. *Current Biology*.

54. S. Gavrilets, J. B. Losos, Adaptive Radiation: Contrasting Theory with Data. *Science* **323**, 732-737 (2009).

55. T. Ingram, L. J. Harmon, J. B. Shurin, When should we expect early bursts of trait evolution in comparative data? Predictions from an evolutionary food web model. *Journal of Evolutionary Biology* **25**, 1902-1910 (2012).

56. L. J. Harmon, J. B. Losos, T. Jonathan Davies, R. G. Gillespie, J. L. Gittleman, W. Bryan Jennings, K. H. Kozak, M. A. McPeek, F. Moreno-Roark, T. J. Near, A. Purvis, R. E. Ricklefs, D. Schluter, J. A. Schulte Ii, O. Seehausen, B. L. Sidlauskas, O. Torres-Carvajal, J. T. Weir, A. Ø. Mooers, Early bursts of body size and shape evolution are rare in comparative data. *Evolution* **64**, 2385-2396 (2010).

57. P. J. Wagner, Early bursts of disparity and the reorganization of character integration. *Proceedings of the Royal Society B: Biological Sciences* **285**, 20181604 (2018).

58. M. Hughes, S. Gerber, M. A. Wills, Clades reach highest morphological disparity early in their evolution. *Proceedings of the National Academy of Sciences* **110**, 13875-13879 (2013).

59. J. P. Bird, R. Martin, H. R. Akçakaya, J. Gilroy, I. J. Burfield, S. T. Garnett, A. Symes, J. Taylor, Ç. H. Şekercioğlu, S. H. M. Butchart, Generation lengths of the world's birds and their implications for extinction risk. *Conservation Biology* **34**, 1252-1261 (2020).

60. D. T. Ksepka, A. M. Balanoff, N. A. Smith, G. S. Bever, B.-A. S. Bhullar, E. Bourdon, E. L. Braun, J. G. Burleigh, J. A. Clarke, M. W. Colbert, J. R. Corfield, F. J. Degrange, V. L. De Pietri, C. M. Early, D. J. Field, P. M. Gignac, M. E. L. Gold, R. T. Kimball, S. Kawabe, L. Lefebvre, J. Marugán-Lobón, C. S. Mongle, A. Morhardt, M. A. Norell, R. C. Ridgely, R. S. Rothman, R. P. Scofield, C. P. Tambussi, C. R. Torres, M. van Tuinen, S. A. Walsh, A. Watanabe, L. M. Witmer, A. K. Wright, L. E. Zanno, E. D. Jarvis, J. B. Smaers, Tempo and Pattern of Avian Brain Size Evolution. *Current Biology* **30**, 2026-2036.e2023 (2020).

61. S. C. Stearns, The Influence of Size and Phylogeny on Patterns of Covariation among Life-History Traits in the Mammals. *Oikos* **41**, 173-187 (1983).

62. C. Parins-Fukuchi, Mosaic evolution, preadaptation, and the evolution of evolvability in apes. *Evolution* **74**, 297-310 (2020).

63. J. Romiguier, V. Ranwez, E. J. P. Douzery, N. Galtier, Contrasting GC-content dynamics across 33 mammalian genomes: Relationship with life-history traits and chromosome sizes. *Genome Research* **20**, 1001-1009 (2010).

64. P. Bolívar, L. Guéguen, L. Duret, H. Ellegren, C. F. Mugal, GC-biased gene conversion conceals the prediction of the nearly neutral theory in avian genomes. *Genome Biology* **20**, 5 (2019).

65. S. Singhal, E. M. Leffler, K. Sannareddy, I. Turner, O. Venn, D. M. Hooper, A. I. Strand, Q. Li, B. Raney, C. N. Balakrishnan, S. C. Griffith, G. McVean, M. Przeworski, Stable recombination hotspots in birds. *Science* **350**, 928-932 (2015).

66. S. Reddy, R. T. Kimball, A. Pandey, P. A. Hosner, M. J. Braun, S. J. Hackett, K.-L. Han, J. Harshman, C. J. Huddleston, S. Kingston, B. D. Marks, K. J. Miglia, W. S. Moore, F. H. Sheldon, C. C. Witt, T. Yuri, E. L. Braun, Why Do Phylogenomic Data Sets Yield Conflicting Trees? Data Type Influences the Avian Tree of Life more than Taxon Sampling. *Systematic Biology* **66**, 857-879 (2017).

67. E. L. Braun, R. T. Kimball, Data Types and the Phylogeny of Neoaves. *Birds* **2**, (2021).

68. H. Kuhl, C. Frankl-Vilches, A. Bakker, G. Mayr, G. Nikolaus, S. T. Boerno, S. Klages, B. Timmermann, M. Gahr, An Unbiased Molecular Approach Using 3′-UTRs Resolves the Avian Family-Level Tree of Life. *Molecular Biology and Evolution* **38**, 108-127 (2020).

69. A. R. Lemmon, S. A. Emme, E. M. Lemmon, Anchored Hybrid Enrichment for Massively High-Throughput Phylogenomics. *Systematic Biology* **61**, 727-744 (2012).

70. A. M. Bolger, M. Lohse, B. Usadel, Trimmomatic: a flexible trimmer for Illumina sequence data. *Bioinformatics* **30**, 2114-2120 (2014).

71. T. Magoč, S. L. Salzberg, FLASH: fast length adjustment of short reads to improve genome assemblies. *Bioinformatics* **27**, 2957-2963 (2011).

72. M. G. Grabherr, B. J. Haas, M. Yassour, J. Z. Levin, D. A. Thompson, I. Amit, X. Adiconis, L. Fan, R. Raychowdhury, Q. Zeng, Z. Chen, E. Mauceli, N. Hacohen, A. Gnirke, N. Rhind, F. di Palma, B. W. Birren, C. Nusbaum, K. Lindblad-Toh, N. Friedman, A. Regev, Full-length transcriptome assembly from RNA-Seq data without a reference genome. *Nature Biotechnology* **29**, 644-652 (2011).

73. W. J. Kent, BLAT—The BLAST-Like Alignment Tool. *Genome Research* **12**, 656-664 (2002).

74. H. Li, Aligning sequence reads, clone sequences and assembly contigs with BWA-MEM. *arXiv preprint arXiv:1303.3997,* (2013).

75. G. A. Van der Auwera, B. D. O'Connor, *Genomics in the Cloud: Using Docker, GATK, and WDL in Terra* (O'Reilly Media, Incorporated, 2020).

76. V. Ranwez, N. Chantret, F. Delsuc, "Aligning Protein-Coding Nucleotide Sequences with MACSE" in *Multiple Sequence Alignment: Methods and Protocols,* K. Katoh, Ed. (Springer US, New York, NY, 2021), pp. 51-70.

77. A. Di Franco, R. Poujol, D. Baurain, H. Philippe, Evaluating the usefulness of alignment filtering methods to reduce the impact of errors on evolutionary inferences. *BMC Evolutionary Biology* **19**, 21 (2019).

78. R. K. Bradley, A. Roberts, M. Smoot, S. Juvekar, J. Do, C. Dewey, I. Holmes, L. Pachter, Fast Statistical Alignment. *PLOS Computational Biology* **5**, e1000392 (2009).

79. M. L. Borowiec, AMAS: a fast tool for alignment manipulation and computing of summary statistics. *PeerJ* **4**, e1660 (2016).

80. S. Capella-Gutiérrez, J. M. Silla-Martínez, T. Gabaldón, trimAl: a tool for automated alignment trimming in large-scale phylogenetic analyses. *Bioinformatics* **25**, 1972-1973 (2009).

81. G. Talavera, J. Castresana, Improvement of Phylogenies after Removing Divergent and Ambiguously Aligned Blocks from Protein Sequence Alignments. *Systematic Biology* **56**, 564-577 (2007).

82. G. Tan, M. Muffato, C. Ledergerber, J. Herrero, N. Goldman, M. Gil, C. Dessimoz, Current Methods for Automated Filtering of Multiple Sequence Alignments Frequently Worsen Single-Gene Phylogenetic Inference. *Systematic Biology* **64**, 778-791 (2015).

83. R. Allio, A. Schomaker-Bastos, J. Romiguier, F. Prosdocimi, B. Nabholz, F. Delsuc, MitoFinder: Efficient automated large-scale extraction of mitogenomic data in target enrichment phylogenomics. *Molecular Ecology Resources* **20**, 892-905 (2020).

84. J. M. McCullough, R. G. Moyle, B. T. Smith, M. J. Andersen, A Laurasian origin for a pantropical bird radiation is supported by genomic and fossil data (Aves: Coraciiformes). *Proceedings of the Royal Society B: Biological Sciences* **286**, 20190122 (2019).

85. P. G. P. Ericson, M. Irestedt, J. A. A. Nylander, L. Christidis, L. Joseph, Y. Qu, Parallel Evolution of Bower-Building Behavior in Two Groups of Bowerbirds Suggested by Phylogenomics. *Systematic Biology* **69**, 820-829 (2020).

86. R. T. Kimball, M. Guido, P. A. Hosner, E. L. Braun, When good mitochondria go bad: Cyto-nuclear discordance in landfowl (Aves: Galliformes). *Gene* **801**, 145841 (2021).

87. M. G. Harvey, G. A. Bravo, S. Claramunt, A. M. Cuervo, G. E. Derryberry, J. Battilana, G. F. Seeholzer, J. S. McKay, B. C. O'Meara, B. C. Faircloth, S. V. Edwards, J. Pérez-Emán, R. G. Moyle, F. H. Sheldon, A. Aleixo, B. T. Smith, R. T. Chesser, L. F. Silveira, J. Cracraft, R. T. Brumfield, E. P. Derryberry, The evolution of a tropical biodiversity hotspot. *Science* **370**, 1343-1348 (2020).

88. B. T. Smith, J. Merwin, K. L. Provost, G. Thom, R. T. Brumfield, M. Ferreira, W. M. Mauck III, R. G. Moyle, T. Wright, L. Joseph, Phylogenomic analysis of the parrots of the world distinguishes artifactual from biological sources of gene tree discordance. *Systematic Biology,* (2022).

89. D. M. Standley, K. Katoh, MAFFT Multiple Sequence Alignment Software Version 7: Improvements in Performance and Usability. *Molecular Biology and Evolution* **30**, 772-780 (2013).

90. B. Q. Minh, H. A. Schmidt, O. Chernomor, D. Schrempf, M. D. Woodhams, A. von Haeseler, R. Lanfear, IQ-TREE 2: New Models and Efficient Methods for Phylogenetic Inference in the Genomic Era. *Molecular Biology and Evolution* **37**, 1530-1534 (2020).

91. D. T. Hoang, L. S. Vinh, O. Chernomor, B. Q. Minh, A. von Haeseler, UFBoot2: Improving the Ultrafast Bootstrap Approximation. *Molecular Biology and Evolution* **35**, 518-522 (2018).

92. A. Suh, The phylogenomic forest of bird trees contains a hard polytomy at the root of Neoaves. *Zoologica Scripta* **45**, 50-62 (2016).

93. L.-T. Nguyen, H. A. Schmidt, A. von Haeseler, B. Q. Minh, IQ-TREE: A Fast and Effective Stochastic Algorithm for Estimating Maximum-Likelihood Phylogenies. *Molecular Biology and Evolution* **32**, 268-274 (2014).

94. S. Kalyaanamoorthy, B. Q. Minh, T. K. F. Wong, A. von Haeseler, L. S. Jermiin, ModelFinder: fast model selection for accurate phylogenetic estimates. *Nature Methods* **14**, 587 (2017).

95. O. Chernomor, B. Q. Minh, A. von Haeseler, Terrace Aware Data Structure for Phylogenomic Inference from Supermatrices. *Systematic Biology* **65**, 997-1008 (2016).

96. J. M. Eastman, L. J. Harmon, D. C. Tank, Congruification: support for time scaling large phylogenetic trees. *Methods in Ecology and Evolution* **4**, 688-691 (2013).

97. S. A. Smith, B. C. O'Meara, treePL: divergence time estimation using penalized likelihood for large phylogenies. *Bioinformatics* **28**, 2689-2690 (2012).

98. M. E. Alfaro, F. Santini, C. Brock, H. Alamillo, A. Dornburg, D. L. Rabosky, G. Carnevale, L. J. Harmon, Nine exceptional radiations plus high turnover explain species diversity in jawed vertebrates. *Proceedings of the National Academy of Sciences* **106**, 13410-13414 (2009).

99. V. Mitov, K. Bartoszek, T. Stadler, Automatic generation of evolutionary hypotheses using mixed Gaussian phylogenetic models. *Proceedings of the National Academy of Sciences* **116**, 16921-16926 (2019).

100. G. Schwarz, Estimating the dimension of a model. *The annals of statistics* **6**, 461-464 (1978).

101. N. Ly-Trong, S. Naser-Khdour, R. Lanfear, B. Q. Minh, AliSim: A Fast and Versatile Phylogenetic Sequence Simulator for the Genomic Era. *Molecular Biology and Evolution* **39**, (2022).

102. Y. Benjamini, Y. Hochberg, Controlling the False Discovery Rate: A Practical and Powerful Approach to Multiple Testing. *Journal of the Royal Statistical Society: Series B (Methodological)* **57**, 289-300 (1995).

103. L. s. Tung Ho, C. Ané, A Linear-Time Algorithm for Gaussian and Non-Gaussian Trait Evolution Models. *Systematic Biology* **63**, 397-408 (2014).

104. A. R. Ives, T. Garland, Jr., Phylogenetic Logistic Regression for Binary Dependent Variables. *Systematic Biology* **59**, 9-26 (2010).

105. E. Paradis, J. Claude, Analysis of Comparative Data Using Generalized Estimating Equations. *Journal of Theoretical Biology* **218**, 175-185 (2002).

106. L. Harmon. Phylogenetic comparative methods: learning from trees. (2018); https:// lukejharmon.github.io/pcm/.

107. C. Ané, J. Berv, Ed. (github, https://github.com/lamho86/phylolm/issues/46, 2021).

108. C. Parins-Fukuchi, G. W. Stull, S. A. Smith, Phylogenomic conflict coincides with rapid morphological innovation. *Proceedings of the National Academy of Sciences* **118**, e2023058118 (2021).

109. F. K. Mendes, M. W. Hahn, Gene Tree Discordance Causes Apparent Substitution Rate Variation. *Systematic Biology*, (2016).

110. B. Q. Minh, M. A. T. Nguyen, A. von Haeseler, Ultrafast Approximation for Phylogenetic Bootstrap. *Molecular Biology and Evolution* **30**, 1188-1195 (2013).

111. Y. Clément, G. Sarah, Y. Holtz, F. Homa, S. Pointet, S. Contreras, B. Nabholz, F. Sabot, L. Sauné, M. Ardisson, R. Bacilieri, G. Besnard, A. Berger, C. Cardi, F. De Bellis, O. Fouet, C. Jourda, B. Khadari, C. Lanaud, T. Leroy, D. Pot, C. Sauvage, N. Scarcelli, J. Tregear, Y. Vigouroux, N. Yahiaoui, M. Ruiz, S. Santoni, J.-P. Labouisse, J.-L. Pham, J. David, S. Glémin,

Evolutionary forces affecting synonymous variations in plant genomes. *PLOS Genetics* **13**, e1006799 (2017).

112. S. Qiu, R. Bergero, K. Zeng, D. Charlesworth, Patterns of Codon Usage Bias in Silene latifolia. *Molecular Biology and Evolution* **28**, 771-780 (2010).

113. X.-F. Wan, D. Xu, A. Kleinhofs, J. Zhou, Quantitative relationship between synonymous codon usage bias and GC composition across unicellular genomes. *BMC Evolutionary Biology* **4**, 19 (2004).

114. F. Wright, The ‘effective number of codons’ used in a gene. *Gene* **87**, 23-29 (1990).

115. J. A. Novembre, Accounting for Background Nucleotide Composition When Measuring Codon Usage Bias. *Molecular Biology and Evolution* **19**, 1390-1394 (2002).

116. C. E. Shannon, A mathematical theory of communication. *The Bell system technical journal* **27**, 379-423 (1948).

117. L. J. Harmon, J. T. Weir, C. D. Brock, R. E. Glor, W. Challenger, GEIGER: investigating evolutionary radiations. *Bioinformatics* **24**, 129-131 (2008).

118. L. J. Revell, phytools: an R package for phylogenetic comparative biology (and other things). *Methods in Ecology and Evolution* **3**, 217-223 (2012).

119. T. Garland, Jr., A. W. Dickerman, C. M. Janis, J. A. Jones, Phylogenetic Analysis of Covariance by Computer Simulation. *Systematic Biology* **42**, 265-292 (1993).

120. T. E. Martin, Avian Life-History Evolution has an Eminent Past: Does it Have a Bright Future? *The Auk* **121**, 289-301 (2004).

121. F. J. Ballesteros, V. J. Martinez, B. Luque, L. Lacasa, E. Valor, A. Moya, On the thermodynamic origin of metabolic scaling. *Scientific Reports* **8**, 1448 (2018).

122. J. H. Brown, J. F. Gillooly, A. P. Allen, V. M. Savage, G. B. West, TOWARD A METABOLIC THEORY OF ECOLOGY. *Ecology* **85**, 1771-1789 (2004).

123. G. B. West, J. H. Brown, B. J. Enquist, A General Model for the Origin of Allometric Scaling Laws in Biology. *Science* **276**, 122-126 (1997).

124. A. J. Hulbert, A Sceptics View: “Kleiber’s Law” or the “3/4 Rule” is neither a Law nor a Rule but Rather an Empirical Approximation. *Systems* **2**, 186-202 (2014).

125. R. Tacutu, T. Craig, A. Budovsky, D. Wuttke, G. Lehmann, D. Taranukha, J. Costa, V. E. Fraifeld, J. P. de Magalhães, Human Ageing Genomic Resources: Integrated databases and tools for the biology and genetics of ageing. *Nucleic Acids Research* **41**, D1027-D1033 (2013).

126. E. W. Goolsby, J. Bruggeman, C. Ané, Rphylopars: fast multivariate phylogenetic comparative methods for missing data and within-species variation. *Methods in Ecology and Evolution* **8**, 22-27 (2017).

127. J. Clavel, G. Escarguel, G. Merceron, mvmorph: an r package for fitting multivariate evolutionary models to morphometric data. *Methods in Ecology and Evolution* **6**, 1311-1319 (2015).

128. J. C. Uyeda, L. J. Harmon, A Novel Bayesian Method for Inferring and Interpreting the Dynamics of Adaptive Landscapes from Phylogenetic Comparative Data. *Systematic Biology* **63**, 902-918 (2014).

129. P. Bastide, C. Ané, S. Robin, M. Mariadassou, Inference of Adaptive Shifts for Multivariate Correlated Traits. *Systematic Biology* **67**, 662-680 (2018).

130. D. S. Caetano, L. J. Harmon, ratematrix: An R package for studying evolutionary integration among several traits on phylogenetic trees. *Methods in Ecology and Evolution* **8**, 1920-1927 (2017).

131. R. A. Fisher, *Statistical methods for research workers, 5th ed* (Statistical methods for research workers, 5th ed., Edinburgh, Oliver and Boyd, 1934).

132. M. Kuhn, H. Wickham, Tidymodels: a collection of packages for modeling and machine learning using tidyverse principles. (2020).

133. RR Core Team, R: A Language and Environment for Statistical Computing. R Foundation for Statistical Computing, (Vienna, 2018); https://R-project.org.

134. T. Nagler, A generic approach to nonparametric function estimation with mixed data. *Statistics & Probability Letters* **137**, 326-330 (2018).

135. L. Breiman, Random Forests. *Machine Learning* **45**, 5-32 (2001).

136. H. Tin Kam, in *Proceedings of 3rd International Conference on Document Analysis and Recognition*. (1995), vol. 1, pp. 278-282 vol.271.

137. J. Fürnkranz, "Decision Tree" in *Encyclopedia of Machine Learning,* C. Sammut, G. I. Webb, Eds. (Springer US, Boston, MA, 2010), pp. 263-267.

138. T. C. D. Lucas, A translucent box: interpretable machine learning in ecology. *Ecological Monographs* **90**, e01422 (2020).

139. M. Benito, spatialRF: Easy spatial regression with random forest. *R package version 1.1.0* **1**, (2021).

140. H. Haibo, B. Yang, E. A. Garcia, L. Shutao, in *2008 IEEE International Joint Conference on Neural Networks (IEEE World Congress on Computational Intelligence)*. (2008), pp. 1322-1328.

141. A. Liaw, M. Wiener, Classification and regression by randomForest. *R news* **2**, 18-22 (2002).

142. D. J. Hand, R. J. Till, A Simple Generalisation of the Area Under the ROC Curve for Multiple Class Classification Problems. *Machine Learning* **45**, 171-186 (2001).

143. C. Molnar. (christophm.github.io/interpretable-ml-book/, 2022).

144. B. M. Greenwell, B. C. Boehmke, B. Gray, Variable Importance Plots-An Introduction to the vip Package. *R J.* **12**, 343 (2020).

145. A. Gelman, D. B. Rubin, Inference from iterative simulation using multiple sequences. *Statistical science*, 457-472 (1992).

146. S. P. Brooks, A. Gelman, General methods for monitoring convergence of iterative simulations. *Journal of computational and graphical statistics* **7**, 434-455 (1998).

147. M. Brevet, N. Lartillot, Reconstructing the History of Variation in Effective Population Size along Phylogenies. *Genome Biology and Evolution* **13**, (2021).

148. John Damuth, A Macroevolutionary Explanation for Energy Equivalence in the Scaling of Body Size and Population Density. *The American Naturalist* **169**, 621-631 (2007).

149. D. J. Currie, What Shape Is the Relationship between Body Size and Population Density? *Oikos* **66**, 353-358 (1993).

150. J. G. Wagler, *Natürliches System der Amphibien: mit vorangehender Classification der Säugethiere und Vögel : ein Beitrag zur vergleichenden Zoologie* (J.G. Cotta, 1830).

151. J. C. Uyeda, D. S. Caetano, M. W. Pennell, Comparative Analysis of Principal Components Can be Misleading. *Systematic Biology* **64**, 677-689 (2015).

152. C. v. Linné, L. Salvius, *Caroli Linnaei...Systema naturae per regna tria naturae :secundum classes, ordines, genera, species, cum characteribus, differentiis, synonymis, locis* (Impensis Direct. Laurentii Salvii, Holmiae, 1758), vol. v.1.

153. W. P. Pycraft, L. W. R. B. Rothschild, *A monograph of the genus Casuarius* (Zoological Society of London, London, 1900).

154. T. Yuri, R. T. Kimball, J. Harshman, R. C. K. Bowie, M. J. Braun, J. L. Chojnowski, K.-L. Han, S. J. Hackett, C. J. Huddleston, W. S. Moore, S. Reddy, F. H. Sheldon, D. W. Steadman, C. C. Witt, E. L. Braun, Parsimony and model-based analyses of indels in avian nuclear genes reveal congruent and incongruent phylogenetic signals. *Biology* **2**, 419-444. (2013).

155. T. H. Huxley, *A Manual of the Anatomy of Vertebrated Animals* (D. Appleton, 1872).

156. C. G. Sibley, J. E. Ahlquist, *Phylogeny and classification of birds: a study in molecular evolution* (Yale University Press, 1990).
